# Prevalence and Molecular Evolution of Parvovirus in Cats in Eastern Shandong, China, between 2021 and 2022

**DOI:** 10.1155/2024/5514806

**Published:** 2024-01-05

**Authors:** Jingyu Wang, Zhirong Yan, Haoran Liu, Wenjie Wang, Yakun Liu, Xu Zhu, Lili Tian, Jianjun Zhao, Qisheng Peng, Zhenwei Bi

**Affiliations:** ^1^State Key Laboratory for Diagnosis and Treatment of Severe Zoonotic Infectious Diseases, Key Laboratory for Zoonosis Research of the Ministry of Education, Institute of Zoonosis and College of Veterinary Medicine, Jilin University, Changchun 130062, China; ^2^Institute of Veterinary Medicine, Key Laboratory of Veterinary Biological Engineering and Technology, Ministry of Agriculture and Rural Affairs, Jiangsu Academy of Agricultural Sciences, Nanjing, Jiangsu 210014, China; ^3^School of Pet Science and Technology, Jiangsu Agri-Animal Husbandry Vocational College, Taizhou, Jiangsu 225300, China; ^4^College of Animal Science and Veterinary Medicine, Heilongjiang Bayi Agricultural University, Daqing, Heilongjiang, China; ^5^Shanghai GlinX Biotechnology Company Limited, Shanghai 200050, China; ^6^Department of Modern Agriculture, Linyi Vocational University of Science and Technology, Linyi, Shandong 276025, China; ^7^GuoTai (Taizhou) Center of Technology Innovation for Veterinary Biologicals, Taizhou, Jiangsu 225300, China

## Abstract

Feline panleukopenia (FPL) is a highly contagious infectious disease caused by infection with feline parvovirus (FPV) and canine parvovirus type 2 (CPV-2). In recent years, the number of cats with FPL has increased with the expansion of pet cat population in China. The feces of 51 cats with diarrhea symptoms collected from 2021 to 2022 in Eastern Shandong, China, were detected by polymerase chain reaction for parvovirus and other viruses related to feline diarrhea to investigate the prevalence and gene variation of parvovirus in cats. In all the 51 samples, 45.1% (23/51) were positive for at least one viral pathogen, and the positivity of parvovirus was 41.2% (21/51), showing a high prevalence. Multiple-pathogen testing indicated high-coinfection rates of 42.9% (9/21) with other common viruses in parvovirus-positive cats. Most of the coinfections are feline coronavirus (FCoV), followed by feline astrovirus (FAstV) and feline bocavirus (FBoV). The complete VP2 sequences of 21 parvoviruses were obtained. Among them, 20 sequences were identified as FPV, and only one was CPV-2c of Asian origin, which was first detected from cats in Eastern Shandong, China. A phylogenetic tree of the 20 FPVs was constructed together with 698 FPVs (cat/dog host) worldwide on the basis of complete VP2. The 18 FPVs displayed high-sequence identity to one another (99.8%–100%), and they were clustered into FPV-G1 group, whereas the other two were clustered into FPV-G3 group. The FPV-G1 group increased dramatically to become predominant after 2019 in China, contributing to the prevalence of A91S mutation due to 96.07% FPV-G1 with A91S mutation as well as 100% of FPV-G2 and 99.12% of FPV-G3 with 91A in the statistical analysis. This study enriched the understanding of the prevalence, molecular evolution, and cross-species transmission of parvovirus in cats and provided a basis for responding to challenges in the diagnosis and treatment of FPL.

## 1. Introduction

Feline panleukopenia (FPL) is a highly contagious infectious disease related to feline diarrhea [1]. Cats with this disease usually present with severe leukopenia, vomiting, diarrhea, dehydration, and even systemic symptoms. The disease often occurs, with higher mortality, in young cats [2], which have a higher mitotic index in intestinal crypt cells [3]. Even in countries with high vaccination rates, sudden epidemics of FPL often occur [4].

FPL is mainly caused not only by feline parvovirus (FPV) but also a small number of canine parvovirus type 2 (CPV-2) [2]. They are small nonenveloped linear ssDNA viruses, members of the family *Parvoviridae*, subfamily *Parvovirinae*, and genus *Protoparvovirus*; and recently included in the unique species Carnivore protoparvovirus 1. VP2 is the major structural protein of FPV and CPV-2. It induces the production of protective antibodies and is responsible for receptor binding, host range, hemagglutination, pathogenicity, and virulence of the virus [2, 5–8]. The evolution of FPV was mainly forced by random genetic drift [9]. Since FPV was first identified in 1964 [10], it has not evolved into genotype, and no significant changes have occurred in its antigenic and biological properties. In 1978, CPV-2 was first identified from dogs, and it was considered to be a change in host range due to mutations in FPV. The key amino-acid residues (80R, 93N, 103A, 323N, 564S, and 568G) in VP2 protein has been widely used to distinguish CPV-2 from FPV [11]. CPV-2 is divided into different genotypes on the basis of the key amino-acid residues of VP2 protein. Original CPV-2 and CPV-2a have six amino-acid differences (M87L, I101T, A300G, D305Y, N375D, and V555I). Compared with CPV-2a, CPV-2b had only one N426D mutation. The new CPV-2a included 426N and 297A, the new CPV-2b included 426D and 297A, and the CPV-2c included 426E and 297A [12]. The variants of CPV-2 have been detected in cat diarrhea samples [2, 11] and acquired the ability to infect feline hosts [3, 6, 13]. FPV was also detected in canine diarrhea samples occasionally [14]. These studies suggest that different genotypes of CPV-2 and FPV have different frequencies of interspecific transmission between dogs and cats [15–17]. In addition, genetic recombination plays an important role in the evolution of FPV and CPV-2 [18, 15–17]. It brings difficulties to the prevention and control of parvovirus. Coinfection of FPV with some other enteric viruses has been reported in cat diarrhea samples [19], such as bocaparvovirus, chaphamaparvovirus, astrovirus, coronavirus, calicivirus, and kobuvirus [12, 20–23] which may aggravate diarrhea symptoms in cats, thus also presenting difficulties in the prevention and treatment of FPL.

The cases of diarrhea in cats caused by parvovirus increased in Qingdao and Yantai of Shandong, China, from the end of 2021 to the beginning of 2022, although some cats have been immunized. Stool samples from 51 cases of feline diarrhea were collected from Qingdao and Yantai from 2021 to 2022 in this study, and parvovirus and diarrhea-associated viruses were detected and the molecular evolution of VP2 of parvovirus from cat was analyzed to gain insights into the local prevalence and molecular characterization of parvovirus and diarrhea-associated viruses in cats in China.

## 2. Materials and Methods

### 2.1. Clinical Sample Collection

A total of 51 fecal swabs were collected from cats with diarrhea, vomiting, fever, or dehydration in Yantai (*n* = 31) and Qingdao (*n* = 20) in Eastern Shandong Province, China, from March 2021 to June 2022. Age, breed, and vaccination status were not recorded.

### 2.2. DNA/RNA Extraction from Fecal Swabs

Total viral DNA was extracted from 200 *µ*L fecal supernatants by using the Genomic DNA Viral Genome Extraction Kit (Solarbio Technology Co., Ltd., Beijing, China) and total viral RNA was extracted from 500 *µ*L fecal supernatants by using TRIzol reagent (TaKaRa, Dalian, China) in accordance with the manufacturer's instructions. RNA was transcribed to cDNA by using the ABScriptⅢ RT Master Mix with gDNA remover kit (ABclonal). The extracted DNA and transcribed cDNA were stored at −40°C.

### 2.3. Polymerase Chain Reaction (PCR)

All clinical samples were examined for FPV and other important enteric viruses, including feline bocavirus (FBoV), feline bufaviruses (FBuV), feline chaphamaparvovirus (FChPV), feline stool-associated circular virus (FSCV), feline enteric coronaviruses (FCoV), feline calicivirus (FCV), feline kobuvirus (FKoV), feline noroviruses (FNoV), feline astroviruses (FAstV), and feline rotavirus (FRV). Specific primers were designed for the amplification of the above viruses and synthesized by the Sangong Bioengineering Technology Service Co., Ltd. (Shanghai, China). The specific primers and expected amplified fragments are shown in *Supplementary 1* [23–30]. The PCR reaction contained 1 *µ*L DNA template, 1 *µ*L of each primer (10 *µ*M), 12.5 *µ*L Premix Taq (Takara Biomedical Technology Co., Ltd., Beijing, China), and 9.5 *µ*L nuclease-free water. The thermal cycling program of FPV PCR reaction consisted of an initial 94°C for 10 min, followed by 35 cycles of 94°C for 30 s, 55°C for 30 s, and 72°C for 1 min, and then a final 72°C for 10 min, other enteric viruses were detected by touchdown PCR reactions with annealing temperatures decreasing from 60°C to 53°C during 35 cycles. The PCR products were connected with pMD 19-T vector (Takara Biomedical Technology Co., Ltd., Beijing, China) or M5 HiPer Ptopo-TA Clong Kit TA (Juhemei Biotechnology Co., Ltd., Beijing, China), which were transformed into *Escherichia coli* DH5*α* competent cells (Takara Biomedical Technology Co., Ltd., Beijing, China). Positive clones were selected by PCR and then sent to the Sangong Bioengineering Technology Service Co., Ltd. (Shanghai, China) for sequencing. The detection of FPV, FCoV, FAstV, FBoV, and FChPV in PCR-positive samples was further verified using feline gastrointestinal tract five-joint kits (InCycle, GlinX Company, China).

### 2.4. Sequence Alignment and Phylogeny Analysis

The complete VP2 of 698 FPVs (cat/dog host) worldwide and 77 CPVs, including 45 CPVs (cat host), were obtained from the GenBank database, as listed in *Supplementary 2*. The MEGA software (version 7.0) was used to construct phylogenetic trees of the complete VP2 of parvovirus, a model test was performed to identify the optimized model and subsequently the phylogenetic relationships were calculated using the maximum likelihood method, with statistical analysis on the basis of 1,000 bootstraps. The nucleotide and deduced amino-acid sequences of the complete VP2 of parvoviruses were aligned using DNASTAR software.

### 2.5. 3D Modeling Prediction of FPV VP2 Protein Structure

To study whether the A91S mutation alters the structural conformation of FPV VP2 protein, FPV-91A SDYT22 (OQ535507), and FPV-91S SDQD6 (OQ535496) were created, respectively, by SWISSMODEL (https://swissmodel.expasy.org), based on the FPV VP2 modeling (1fpv.1.A). The 3D structures were visualized and compared using Pymol.

## 3. Results

### 3.1. Detection and Coinfection Analysis of Parvovirus

From March 2021 to June 2022, a surveillance of viral enteric disease in domestic cats was conducted in Eastern Shandong, China. The 51 feline fecal samples from Yantai (*n* = 31) and Qingdao (*n* = 20) were used to detect parvovirus by PCR, and the positivity was 41.2% (21/51). Further, all samples were examined by RT-PCR/PCR for other important enteric viruses: FBoV, FBuV, FChPV, FSCV, FCoV, FCV, FKoV, FNoV, FAstV, and FRV. The positive samples for FPV, FCoV, FAstV, FBoV, and FChPV were further verified by feline gastrointestinal tract five-joint kits, the results were consistent with those of RT-PCR/PCR. The results showed positive rates of 15.69% (8/51) in FCoV, 9.8% (5/51) in FAstV, 7.8% (4/51) in FBoV, and 2.0% (1/51) in FKoV, which were detected in parvovirus-positive samples, in addition to two samples (SDQD-5 and SDYT-61) that were separately detected for FChPV and FCoV. Thus, the detection rate of the virus in the 51 samples was 45.1% (23/51). Among 21 parvovirus-positive samples, 12 were detected as single-virus infections (57.14%, 12/21), whereas 42.86% (9/21) were coinfected with one or more other viruses as follows: FPV and FCoV (*n* = 2); FPV and FKoV (*n* = 1); FPV and FAstV (*n* = 1); FPV, FBoV, and FCoV (*n* = 1); FPV, FCoV, and FAstV (*n* = 1); and FPV, FBoV, FCoV, and FAstV (*n* = 3). However, FBuV, FSCV, FCV, FNoV, and FRV were not detected in this investigation. These results showed that coinfection should also be fully considered in the clinical diagnosis and treatment of FPL cases. The information of parvovirus and coinfected diarrhea-associated virus were enumerated in *Supplementary 3*.

### 3.2. Characterization of VP2 of FPV and CPV-2

The complete VP2 amplified from 21 cat parvovirus-positive samples were 1,755 bp in length and coded 584 aa. The key amino-acid residues (80R, 93N, 103A, 323N, 564S, and 568G) in VP2 protein have been widely used to distinguish CPV-2 from FPV [11]. Accordingly, the VP2 of SDYT-2 was determined as CPV-2, whereas the other 20 VP2 sequences were determined as FPV. SDYT-2 was considered as CPV-2c genotype due to the existence of 426E and 297A in VP2, which were characterized for CPV-2c in genotype classification of CPV-2 [3]. The F267Y, Y324I, and Q370R mutations served as the evolutionary force that further divided CPV-2c into CPV-2c-Ⅰ (Europe origin) and CPV-2c-Ⅱ (Asian origin) [9]. Thus, SDYT-2 with the three characteristic mutations belonged to CPV-2c-Ⅱ (Table 1).

The amino acid 87 in the VP2 of FPV and or original CPV-2 was M, whereas the variants of CPV-2 (CPV-2a, CPV-2b, new CPV-2a, new CPV-2b, and CPV-2c) were 87L. Statistical analysis revealed that these variants of CPV-2 detected in cat host were also 87L except six sequences: MK65662.1 (new CPV-2a), MK675665.1 (new CPV-2b), MK675665.1 (new CPV-2a), OL547659.1 (CPV-2a), OL547672.1 (new CPV-2a), and OL547676.1 (CPV-2a) with 87M (*Supplementary 2*). In this study, the amino acid 87 of the 20 FPVs was M, whereas the SDYT-2 belonging to CPV-2c was 87L in VP2 (Table 1).

### 3.3. Phylogenic Analysis of Global FPV

As of December 2022, a total of 718 complete VP2 of FPVs (cat/dog hosts) from 22 countries or regions, including 20 FPVs in this study, were registered in GenBank. Among them, 697 FPVs were detected in cat hosts, the remaining 21 FPVs came from dog hosts, and indicating that FPV infection in dogs still accounts for a minority (2.92%, 21/718). The largest number of FPV sequences was found in Asia (437), followed by Europe (137), Oceania (125), South America (1), North America (1), and Africa (3). Most FPV sequences (334) came from China, followed by Australia (116) and Italy (101) (*Supplementary 4*). These results suggest that FPV has been circulating globally.

**Figure 1:**
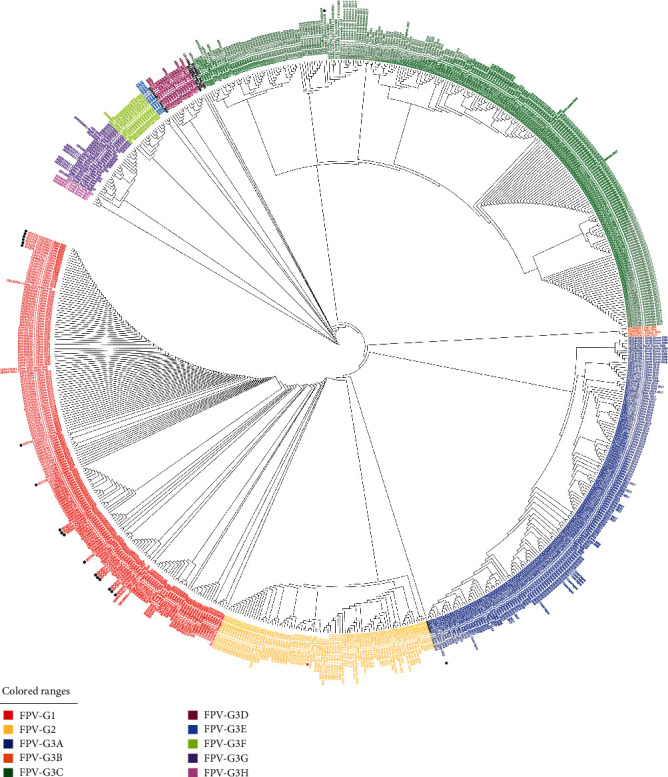
Phylogenetic analysis of complete FPV-VP2 (cat/dog host); black circle indicates the FPV strains in this study, and red square indicates the FPV vaccine strain.

**Figure 2:**
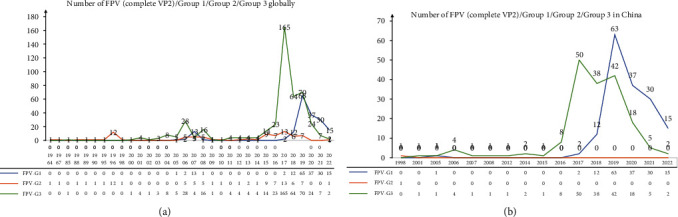
Distribution of different FPV groups. (a) Number of FPV-G1/G2/G3 groups in different years globally. (b) Number of FPV-G1/G2/G3 groups in different years in China.

An FPV phylogenetic tree was constructed based on optimized Tamura 3-parameter model (T92) using the complete VP2 of 20 FPVs obtained in this study, along with 698 reference FPVs (cat/dog hosts) globally retrieved from GenBank. The results showed that 718 VP2 sequences of FPV were divided into three groups: FPV-G1, FPV-G2, and FPV-G3 with FPV-G3A–G3H subgroups (Figure 1). Asia, Europe, and Oceania had large numbers of FPVs in the G3 group, whereas other regions were not summarized due to few sequences (*Supplementary 4*). The VP2 sequences of FPV-G3 accounted for the vast majority (63.65%), which surpassed those of FPV-G1 (24.79%). Few VP2 sequences of FPV-G2 (11.56%), including vaccine strains, were identified (*Supplementary 5*). However, the number of VP2 sequences in the FPV-G1 and FPV-G3 groups was about the same in China (*Supplementary 5*). Several early FPVs in the world were mainly concentrated in the FPV-G2 group containing vaccine strains, and only a complete sequence of the FPV-G2 group was reported in Taiwan, China, in 1998. After 2001, the FPV-G3 group appeared and became dominant from 2015 to 2019 in the world, particularly in China. After 2019, the increased number of G1 group sequences exceeded that of the G3 group in China, which also contributed to the global epidemic trend of the G1 group (Figure 2). G1 strains appeared earlier in China and were rarely reported in the foreign countries; thus, G3 strains remained the dominant strains abroad in the past decade (Figure 2 and *Supplementary 4*).

In this study, the 18 FPVs displayed high-nucleotide sequence identity to one another (99.8%–100%) and were grouped in the FPV-G1 group along with the large Chinese FPV strains. Seven strains (SDQD-6, SDQD-9, SDQD-27, SDQD-28, SDQD-29, SDQD-30, and SDQD-40) shared 100% identity to one another. SDQD-8, SDQD-19, and SDQD-24 have 100% identity, forming a single branch. The 100% homology was found among the five strains (SDQD-12, SDQD-13, SDQD-14, SDQD-15, and SDAD-23). SDQD-21, SDYT-39, and SDYT-41 did not share 100% identity with any sequences but exhibited the close evolution with Chinese strains. The other SDYT-22 and SDYT-1 were FPV-G3 group in the phylogenetic tree. SDYT-1 was clustered into the G3A and closely related to Chinese strains in the subgroup. Interestingly, SDYT-22 shared the close evolution with 26 foreign strains of United Kingdom, Thailand, Egypt, Italy, Portugal, and United Arab Emirates in a branch belonging to the G3C subgroup. No sequence of FPV we detected was clustered into FPV-G2 group containing vaccines. Collectively, 19 of 20 FPVs in Eastern Shandong, China were the same or highly related to that in other parts of China.

### 3.4. Phylogenetic Analysis of Global CPV-2 from Cat Hosts

**Figure 3:**
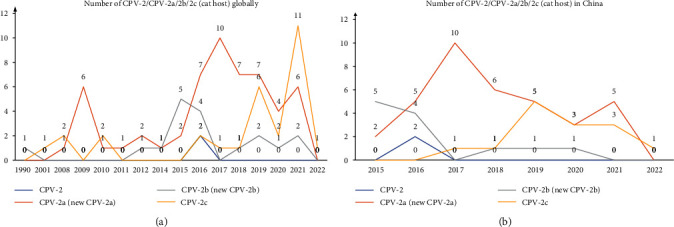
Distribution of different CPV-2 (cat host) and variants. (a) Number of CPV-2/2a/2b/2c (cat host) globally in different years. (b) Number of CPV-2/2a/2b/2c (cat host) in China in different years.

In this study, we analyzed the CPV-2 detected in cat hosts globally. As of December 2022, 45 complete VP2 and 58 partial VP2 of CPV-2 in cat hosts from 13 countries have been registered in GenBank (*Supplementary 2*). The most CPV-2 VP2 sequences came from China (64), followed by India (13), Turkey (11), and Italy (7) (*Supplementary 6*). Asia demonstrated the largest number of CPV-2 VP2 sequences (89), followed by Europe (9), South America (1), North America (1), and Africa (3) (*Supplementary 6*). Among the 104 VP2 sequences from cat hosts, CPV-2a accounted for the vast majority (52.88%), and the proportion of CPV-2c (27.88%) surpassed that of CPV-2b (17.31%). Conversely, only two CPV-2/2-like (1.92%) numbers, which are generally the source of vaccine strains, were identified (*Supplementary 7*). CPV-2a accounted for most sequences in Asia. Other regions did not provide a statistical summary due to small quantity of CPV-2 (*Supplementary 6*). In China, compared with other genotypes of CPV-2, CPV-2a was still the majority (*Supplementary 7*). According to statistics, infection of CPV-2b in cats first appeared in USA in 1990. Other CPV-2 variants (CPV-2a, CPV-2b, and CPV-2c) were subsequently detected in cats globally. Prior to 2020, CPV-2a was the dominant variant in cats. In recent years, the number of CPV-2c in cats has been increasing gradually and replaced CPV-2a as the new dominant variant (Figure 3(a)). Compared the first detection of CPV-2c in Spain in 2001, CPV-2c (MH127909.1) was first reported in Taiwan, China in 2017 and later in the Chinese mainland in cat hosts (Figure 3(b)).

**Figure 4:**
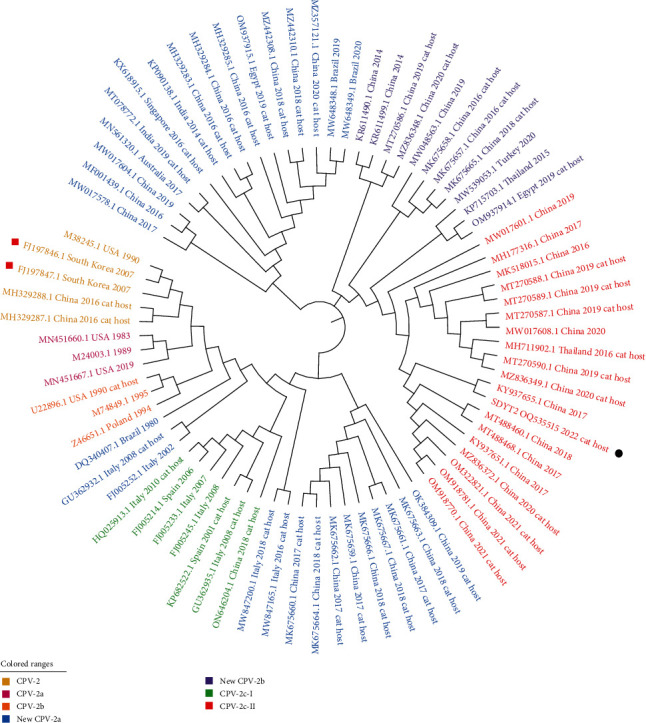
Phylogenetic analysis of complete VP2 of CPV-2; black circle indicates the CPV-2 SDTY-2 sequence in this study, and red square indicates the CPV-2 vaccine strain.

On the basis of complete VP2, the phylogenetic tree was constructed using 46 CPV-2 sequences in cats including SDYT-2 in this study, along with 32 reference CPV-2 sequences in canine hosts of different genotypes retrieved from GenBank (*Supplementary 2*). The 78 CPVs were separated into seven groups: CPV-2, CPV-2a, CPV-2b, new CPV-2a, new CPV-2b, CPV-2c-Ⅰ, and CPV-2c-Ⅱ (Figure 4). SDYT-2 in this study was classified into CPV-2c-Ⅱ genotype, together with 10 Chinese CPV-2 sequences in cats. It displayed the most identity (99.94% nt) with a CPV-2c strain from a dog (KY937655.1) in China within the genotype, which was significantly different from the prevalent CPV-2c-I genotype and the CPV-2 vaccine strains. Interestingly, a CPV-2 (ON646204.1) from cat in China belonged to CPV-2c-Ⅰ. This study reported, for the first time, the detection of CPV-2 c in domestic cats in Yantai, Shandong, China.

### 3.5. Analysis of Specific Amino-Acid Mutation Sites in FPV VP2

**Figure 5:**
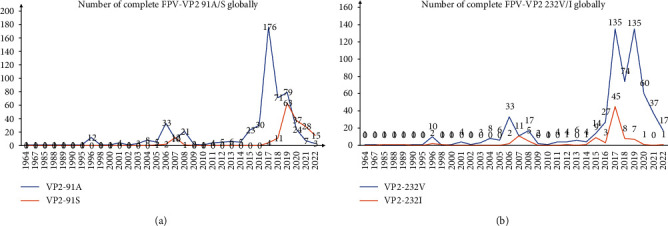
Analysis of global amino-acid mutations at specific sites in complete FPV-VP2 (cat/dog host) gene. (a) Number of amino acids at site 91 of VP2. (b) Number of amino acids at site 232 of VP2.

Chen et al. [13] previously reported the prevalence of A91S mutation of FPV VP2 in China, which may affect the receptor-binding ability of VP2. The substitution of the two amino-acid residues in 91 site was investigated in the 698 complete VP2 of FPVs globally from GenBank as well as 20 FPVs in this study. The results showed 96.07% (171/178) of FPVs in the G1 group was A91S mutation. The exception was observed in five FPVs (EU145593.1, MT270534.1, ON646206.1, ON646207.1, and ON646211.1) with 91A and two FPVs (OM885377.1 and OM918776.1) with A91L mutation. Except for four FPVs (MK671164.1, MK671175.1, ON646208.1, and MK671159.1) that were A91S mutation, 99.12% of FPVs (453/457) in the FPV-G3 group were 91A. All FPV sequences (83/83) of the FPV-G2 group were 91A (*Supplementary 2*). Among the 20 FPVs of the present study, 18 FPVs belonging to the FPV-G1 group were A91S mutation, whereas 91A appeared in the other two SDYT-1 and SDYT-22 of the FPV-G3 group (Table 1). Thus, A91S mutation seems to be dominant in FPV-G1 but not in FPV-G2 and FPV-G3. During 2015–2019, the FPVs with 91A increased due to the global dominance of the FPV-G3 group (Figures 2(a) and 5(a)). After 2019, with the increase in FPVs of the FPV-G1 group in China, the FPV with A91S mutation became dominant (Figure 2(b) and *Supplementary 2*). The V232I mutation probably represented a novel pattern of VP2 genetic evolution on FPV [9, 18]. In this study, all 20 FPVs carried 232V, which remained dominant even though the V232I mutation occurred earlier (Figure 5(b)).

### 3.6. Analysis of Specific Amino-Acid Mutation Sites in CPV-2 VP2

**Figure 6:**
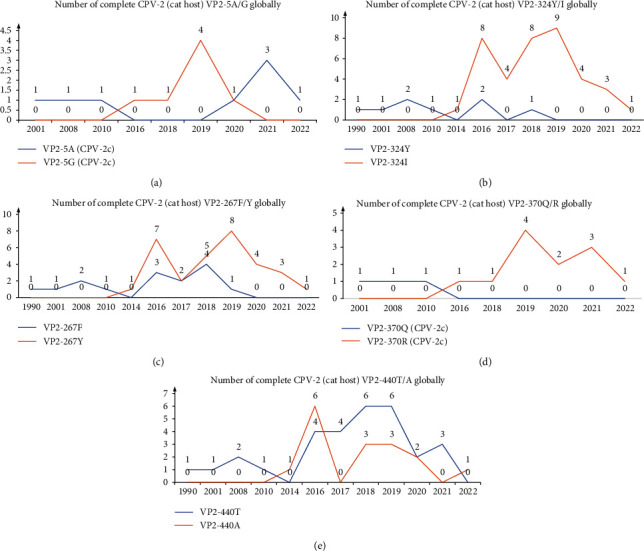
Analysis of global amino-acid mutations at specific sites in complete CPV-2 VP2 (cat host) gene. (a) Number of amino acids at site 5 of VP2 (CPV-2c). (b) Number of amino acids at site 324 of VP2. (c) Number of amino acids at site 267 of VP2. (d) Number of amino acids at site 370 of VP2 (CPV-2c). (e) Number of amino acids at site 440 of VP2.

Hao et al. [31] previously reported the epidemic characteristics of five mutant sites in VP2 (A5G, F267Y, Y324I, Q370R, and T440A). In the present study, the variation in these five sites was monitored in complete VP2 of CPV-2 in cats all over the world. The A5G mutation appeared and only surpassed the original 5A during 2016–2020 (Figure 6(a)). A5S mutation did not occur in CPV-2 SDYT-2 from cats in 2022. A5G mutation often appeared together with F267Y, Y324I, and Q370R mutations in the recently popular Chinese CPV-2c strains [32, 33], which might have had a positive effect on the ability of CPV-2c to infect dogs. SDYT-2 with 5A still had F267Y, Y324I, and Q370R mutations (Figure 6(a)), which were also the characteristic amino acids for defining SDYT-2 as Asian origin of CPV-2c [9]. The frequency of 267Y, 324I, 370R, and 440A mutations became dominant in CPV-2 in cats (Figure 6(b)–6(e)).

### 3.7. The Construction and Analysis of 3D Structure Model of VP2 Protein of A91S Mutation

**Figure 7:**
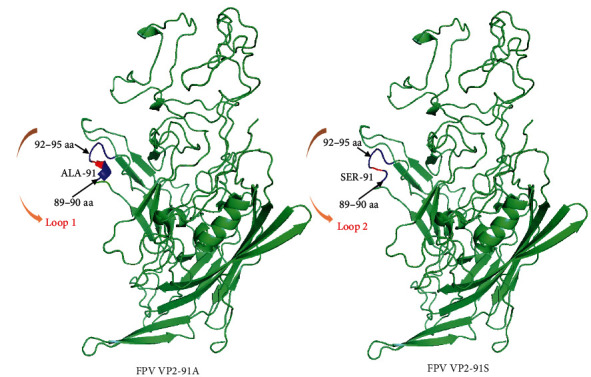
Comparison of the VP2 3D structure of the FPV-91A and FPV-91S variant.

The VP2 3D protein structures of FPV SDYT22 (FPV-91A) and FPV SDQD6 (FPV-91S mutant) were constructed. In prototype FPV-91A model, the amino-acid residues 92–95 of VP2 protein were located on the bulging area of loop 1 forming a random coil structure, while amino-acid residues 89–91 formed an *α*-helix exposed on the surface of VP2 protein. Interestingly, 91 amino-acid mutation in A91S mutant FPV changed the structure of aa 89–91 site from an *α* helix to the random coil, which extended the random coil of amino-acid residues from regions 92–95 in FPV-91A to 89–95 in FPV-91S mutant (Figure 7).

## 4. Discussion

FPV is the main cause of FPL, which is seriously harmful to domestic cats, with high morbidity and mortality. Compared with CPV-2 with different genotypes, FPV has no genotypic subdivisions due to relative conservation in genetic evolution [3, 13, 34]. However, it can be divided into different groups. The number of different FPV strains for grouping in several literatures was limited [9, 35, 36], and the grouping of FPV worldwide must be systematically described. An evolutionary tree was constructed on the basis of the complete VP2 of 718 FPVs, including the 20 FPVs in this study. All FPVs were divided into three major groups, namely, G1–G3, and G3 was further divided into subgroups G3A–G3H. The vast majority (63.65%) of VP2 sequences was FPV-G3, followed by 24.79% of FPV-G1 and 11.56% of FPV-G2 (*Supplementary 5*). However, the number of FPV-G1 and FPV-G3 groups was about the same in China (*Supplementary 5*), because the proportion of the G1 group in China has gradually increased since 2019. The G3 group remained dominant in foreign countries (*Supplementary 5*). The prevalence of A91S mutation has become dominant among FPV strains since 2019 in China [13]. We statistically found that 96.07% of the FPV-G1 group were A91S mutation, whereas 99.12% of the FPV-G3 group and all FPV-G2 were 91A mutation. Thus, the increasing number of FPV-G1 in China since 2019 contributed to the prevalence of A91S mutation. We constructed and compared 3D structure model of VP2 protein of FPV-91A and FPV-91S, showing A91S mutation changed the structure of aa 89–91 site from an *α* helix to the random coil. A recent study analyzed that structure change caused by A91S mutation may affect the receptor-binding ability of VP2 and further influence the biological activities of the A91S FPV variant [13].

Consistent with popular trends in China that FPV-G1 has been become predominant since 2019, 18 of the 20 FPV sequences detected in Eastern Shandong, China, during 2021 and 2022 were clustered into the FPV-G1 group, close to the endemic strains in China. The other two FPVs belonged to FPV-G3 group and one FPV SDYT-22 of them was located in a branch composed of foreign FPVs of FPV-G3C subgroup, which may be transmitted to China from abroad. Unlike in foreign countries, only inactivated cat vaccine (FPV-Cu4) is approved for use in China, which may be one reason that the FPV-G2 of vaccine was not detected in Chinese Mainland.

CPV-2 has evolved into various antigen variants (CPV-2a, CPV-2b, new CPV-2a, new CPV-2b, and CPV-2c) in dogs [5, 37, 38]. Subsequently, these variants were detected in cats and caused the disease of FPL [6, 13, 35, 39]. CPV-2c was first detected in dogs in Italy in 2001 [40]. With the gradual increase in CPV-2c proportion, it has replaced CPV-2a as the new dominant variant in dogs since 2020 worldwide [31]. Our statistics showed the epidemic trend of CPV-2 in cats was similar with that of dogs. In China, the CPV-2c variant was first identified in dogs in 2009; after 2015, reports on CPV-2c in dogs gradually increased [41–43]. From 2018 to now, CPV-2c is the predominant variant in some provinces of China [44, 45]. CPV-2c in cats was first reported in Taiwan, China in 2017 and later in Beijing, Dalian, and Sichuan, China, in 2017–2020 [9, 13, 35]. These Chinese CPV-2c in cats belonged to CPV-2c-Ⅱ (Asian origin), with only one belonging to CPV-2c-Ⅰ (Europe origin), which may be from foreign countries. In this paper, CPV-2c was detected in domestic cats in Yantai, Shandong, China, for the first time. Compared with the 99.32%–99.77% homology with the complete VP2 sequences of CPV-2c detected in cats in China from 2018 to 2021, the SDYT-2 strain showed the highest homology (99.94%) with CPV-2c (KY937655.1) from canine host in Xuzhou, China, in 2017. This finding indicated that the SDYT-2 strain in cat was most likely directly from canine host via cross-species infection rather than the transmission of cat host-derived CPV-2c among cats. In fact, CPV-2 and FPV have showed the different frequencies of interspecific transmission between dogs and cats. The main cause of the FPL is FPV, with only about 5% of FPL being caused by CPV-2 [2]. We statistically found that the proportion of FPVs detected in dogs was 2.92% among all FPV sequences. The gradual emergence of CPV-2c genotype clearly increases the epidemic trend of CPV-2c genotype in cats.

Previous studies reported that the epidemic characteristics of five mutant sites in VP2 (A5G, F267Y, Y324I, Q370R, and T440A) were associated with CPV-2 antigenicity and host range [7, 31, 46]. Among these sites, two mutation sites (A5G and Q370R) have become unique mutations carried by CPV-2c [43]. The trend of the five mutations of CPV-2 in cats is the same with that of CPV-2 from dog hosts. M87L mutation is the key amino-acid site that distinguishes original CPV-2 and FPV from the five variants (CPV-2a, CPV-2b, new CPV-2a, new CPV-2b, and CPV-2c) [47], but 87M was found in two sequences of CPV-2a, three sequences of new CPV-2a, and one sequence of new CPV-2b in cat hosts (*Supplementary 2*). The independent evolution of CPV-2 variants in cats may be worth investigating.

FPV causes local and systemic immunosuppression by damaging gastrointestinal epithelium and bone marrow, and coinfection of FPV with various other enteric viruses has been reported in cats [19, 20, 23, 48, 49]. However, the number and types of coinfected viruses vary in different regions [19, 23, 50–52]. Monitoring cat diarrhea-related viruses in a certain area is important. In the present study, 42.86% (9/21) of cats were coinfected with FPV and other viruses, and FCoV, FAstV and FBoV had a high-coinfection rate with FPV. The three coinfective viruses showed a high-prevalence rate in some regions of China [51, 53, 54]. The detection rate of FChPV was high in clinical cases. It also showed high-coinfection rates with other common viruses in Guangzhou [55]. However, only a single FChPV was detected in the present study. Though no targeted studies have been conducted on the role of the coinfective viruses in enteritis diseases, their positive rate in cats with diarrhea was much higher than that in cats without diarrhea. It suggested that coinfective viruses may be associated with hemorrhagic enteritis in cats [19, 54]. The high coinfection revealed insufficient potency by immunization of current commercial vaccines or by treatment with parvovirus-specific antibodies, which could not give resistance to coinfective viruses, thus bringing challenge to the diagnosis and treatment of FPL.

## 5. Conclusions

The main viruses in cat diarrhea samples in Eastern Shandong from 2021 to 2022 were investigated, and a systematic evolutionary analysis of FPV was performed. FPV-G1 with A91S mutation has become the main dominant in Eastern Shandong and China since 2019, while the FPV-G3 is still dominant abroad. Moreover, the first detection of CPV-2c in cat in Eastern Shandong, China enriches the regional diversity of cross-species transmission of parvovirus between cats and dogs. The high prevalence and coinfection rates of FPV suggested the big challenge for the diagnosis and treatment of FPV. Our data provided a basis for the prevalence and evolution of FPV, demonstrating the urgency in developing a diagnostic method for other coinfected viruses, improving immune strategies, and developing new vaccines.

## Figures and Tables

**Table 1:** Key amino-acid residues in VP2 protein of 21 parvoviruses and reference FPV/CPV-2 strains from GenBank.

GenBank no.	Genotype/group	Key amino acid																				
		5	80	87	91	93	101	103	232	267	297	300	305	323	324	370	375	426	440	555	564	568
OQ535496.1	FPV	A	K	M	S	K	T	V	V	F	S	A	D	D	Y	Q	D	N	T	V	N	A
OQ535497.1	FPV	—	K	M	S	K	T	V	V	F	S	A	D	D	Y	Q	D	N	T	V	—	—
OQ535498.1	FPV	—	K	M	S	K	T	V	V	F	S	A	D	D	Y	Q	D	N	T	V	—	—
OQ535499.1	FPV	—	K	M	S	K	T	V	V	F	S	A	D	D	Y	Q	D	N	T	V	—	—
OQ535500.1	FPV	—	K	M	S	K	T	V	V	F	S	A	D	D	Y	Q	D	N	T	V	N	A
OQ535501.1	FPV	A	K	M	S	K	T	V	V	F	S	A	D	D	Y	Q	D	N	T	V	N	A
OQ535502.1	FPV	—	K	M	S	K	T	V	V	F	S	A	D	D	Y	Q	D	N	T	V	N	A
OQ535503.1	FPV	—	K	M	S	K	T	V	V	F	S	A	D	D	Y	Q	D	N	T	V	—	—
OQ535504.1	FPV	A	K	M	S	K	T	V	V	F	S	A	D	D	Y	Q	D	N	T	V	N	A
OQ535505.1	FPV	A	K	M	S	K	T	V	V	F	S	A	D	D	Y	Q	D	N	T	V	N	A
OQ535506.1	FPV	—	K	M	S	K	T	V	V	F	S	A	D	D	Y	Q	D	N	T	V	—	—
OQ535495.1	FPV	—	K	M	**A**	K	T	V	V	F	S	A	D	D	Y	Q	D	N	T	V	—	—
OQ535507.1	FPV	A	K	M	**A**	K	T	V	V	F	S	A	D	D	Y	Q	D	N	T	V	N	A
OQ535508.1	FPV	—	K	M	S	K	T	V	V	F	S	A	D	D	Y	Q	D	N	T	V	N	—
OQ535509.1	FPV	—	K	M	S	K	T	V	V	F	S	A	D	D	Y	Q	D	N	T	V	—	—
OQ535510.1	FPV	—	K	M	S	K	T	V	V	F	S	A	D	D	Y	Q	D	N	T	V	—	—
OQ535511.1	FPV	—	K	M	S	K	T	V	V	F	S	A	D	D	Y	Q	D	N	T	V	—	—
OQ535512.1	FPV	—	K	M	S	K	T	V	V	F	S	A	D	D	Y	Q	D	N	T	V	—	—
OQ535513.1	FPV	—	K	M	S	K	T	V	V	F	S	A	D	D	Y	Q	D	N	T	V	—	—
OQ535514.1	FPV	—	K	M	S	K	T	V	V	F	S	A	D	D	Y	Q	D	N	T	V	—	—
EU018142.1	FPV	A	K	M	S	K	T	V	I	F	S	A	D	D	Y	Q	D	N	T	V	N	A
EU252146.1	FPV	A	K	M	S	K	T	V	V	F	S	A	D	D	Y	Q	D	N	T	V	N	A
M24004.1	FPV	A	K	M	A	K	T	V	I	F	S	A	D	D	Y	Q	D	N	T	V	N	A
M38246.1 (vaccine)	FPV	A	K	M	A	K	I	V	V	F	S	A	D	D	Y	Q	D	N	T	V	N	A
EU498680.1	FPV	A	K	M	A	K	I	V	I	F	S	A	D	D	Y	Q	D	N	T	V	N	A
ON646205.1	FPV	A	K	M	S	K	T	V	V	F	S	A	D	D	Y	Q	D	N	T	V	N	A
AF015223.1	FPV	A	K	M	A	K	I	V	I	F	S	A	D	D	Y	Q	D	N	T	V	N	A
MK671174.1	FPV	A	K	M	A	K	T	V	V	F	S	A	D	D	Y	Q	D	N	T	V	N	A
MH165482.1	FPV	A	K	M	A	K	T	V	I	F	S	A	D	D	Y	Q	D	N	T	V	N	A
FJ197846.1(vaccine)	CPV-2	A	R	M	A	N	I	A	I	F	S	A	D	N	Y	Q	N	N	T	V	S	G
M38245.1	CPV-2	A	R	M	A	N	I	A	I	F	S	A	D	N	Y	Q	N	N	T	V	S	G
M24003.1	CPV-2a	A	R	L	A	N	T	A	I	F	S	G	Y	N	Y	Q	D	N	T	I	S	G
MN451660.1	CPV-2a	A	R	L	A	N	T	A	I	F	S	G	Y	N	Y	Q	D	N	T	V	S	G
MF001439.1	new CPV-2a	A	R	L	A	N	T	A	I	Y	A	G	Y	N	I	Q	D	N	A	V	S	G
MH329283.1	new CPV-2a	A	R	L	A	N	T	A	I	Y	A	G	Y	N	I	Q	D	N	A	V	S	G
U22896.1	CPV-2b	A	R	L	A	N	T	A	I	F	S	G	Y	N	Y	Q	D	D	T	V	S	G
Z46651.1	CPV-2b	A	R	L	A	N	T	A	I	F	S	G	Y	N	Y	Q	D	D	T	V	S	G
MZ836348.1	new CPV-2b	A	R	L	A	N	T	A	I	Y	A	G	Y	N	I	Q	D	D	A	V	S	G
OM937914.1	new CPV-2b	A	R	L	A	N	T	A	I	Y	A	G	Y	N	I	Q	D	D	A	V	S	G
FJ005245.1	CPV-2c-I	A	R	L	A	N	T	A	I	F	A	G	Y	N	Y	Q	D	E	T	V	S	G
GU362935.1	CPV-2c-I	A	R	L	A	N	T	A	I	F	A	G	Y	N	Y	Q	D	E	T	V	S	G
KP682522.1	CPV-2c-I	A	R	L	A	N	T	A	I	F	A	G	Y	N	Y	Q	D	E	T	V	S	G
ON646204.1	CPV-2c-I	G	R	L	A	N	T	A	I	F	A	G	Y	N	Y	R	D	E	T	V	S	G
MH711902.1	CPV-2c-II	G	R	L	A	N	T	A	I	Y	A	G	Y	N	I	R	D	E	T	V	S	G
MK518015.1	CPV-2c-II	G	R	L	A	N	T	A	I	Y	A	G	Y	N	I	R	D	E	T	V	S	G
MT270587.1	CPV-2c-II	G	R	L	A	N	T	A	I	Y	A	G	Y	N	I	R	D	E	T	V	S	G
MZ836372.1	CPV-2c-II	A	R	L	A	N	T	A	I	Y	A	G	Y	N	I	R	D	E	T	V	S	G
OM322821.1	CPV-2c-II	A	R	L	A	N	T	A	I	Y	A	G	Y	N	I	R	D	E	T	V	S	G
OQ535515.1	CPV-2c-II	A	R	L	A	N	T	A	I	Y	A	G	Y	N	I	R	D	E	T	V	S	G

The FPV and CPV-2 in this study were underlined. The marked bold showed the FPV-VP2 91A in this study.

## Data Availability

All sequences information in this study are from GenBank according to the accession number. The figures and table data used to support the findings of this study are included within the article. The supplementary figures and tables data used to support the findings of this study are included within the supplementary information files.

## Supplementary Materials

Supplementary 1 List of primers used in this study.

Supplementary 2 Key amino-acid residues in VP2 protein of 21 parvoviruses and reference FPV/CPV-2 strains from GenBank.

Supplementary 3 Detection of diarrhea-associated viruses in 51 feline fecal samples. “+” indicates positive test results.

Supplementary 4 Distribution of complete VP2 sequences of FPV (cat/dog host).

Supplementary 5 Quantity proportion of FPV-G1/G2/G3 groups.

Supplementary 6 Distribution of CPV-2 variants (cat host).

Supplementary 7 Quantity proportion of CPV-2/2a/2b/2c (cat host).
