# Supplementary material for: Prevalence and Molecular Evolution of Parvovirus in Cats in Eastern Shandong, China, between 2021 and 2022
Source: Transbound Emerg Dis. 2024 Jan 5;2024:5514806. doi: 10.1155/2024/5514806 (PMC12016963; doi:10.1155/2024/5514806)
Supplement: Supplementary 1 — List of primers used in this study. [file 5514806.f1.docx]

**TableS1**. List of primers used in this study

| **Primer** | **Detection method** | **Sequence 5′ - 3′** | **Location (nt)** | **Purpose** | **size (bp)** | **References** |
| --- | --- | --- | --- | --- | --- | --- |
| FPV1-F | PCR | ATGGTTGGTGACTCTTTGTT | 396-787 | Detection | 392 | This study |
| FPV1-R |  | TACATTTGATTGACACTTCCC |  |  |  |  |
| FBov-F | PCR | TCTACAAGTGGGACATTGGA | 1450-1582 | Detection | 133 | [24] |
| FBov-R |  | GAGCTTGATTGCATTCACGA |  |  |  |  |
| FBuV-F | PCR | CTGGTTTAATCCAGCAGACT | 2872-3074 | Detection | 202 | [25] |
| FBuV-R |  | TGAAGACCAAGGTAGTAGGT |  |  |  |  |
| FChPV-F | PCR | GGTGCGACGACGGAAGATAT | 2185-2516 | Detection | 332 | [21] |
| FChPV-R |  | CAACACCACCATCTCCTGCT |  |  |  |  |
| FSCV-F | PCR | GCTAAGGTCTGCCTCAGGTG | 558-860 | Detection | 303 | [26] |
| FSCV-R |  | CTATGTCCAGGTCGGGAGAA |  |  |  |  |
| FCoV-F | RT-PCR | GATTTGATTTGGCAATGCTAGATTT | 29127-29228 | Detection | 102 | T/CVMA 39 |
| FCoV-R |  | AACAATCACTAGATCCAGACGTTAGCT |  |  |  |  |
| FCV-F | RT-PCR | AACCTGCGCTAACGTGCTT | 5322-6246 | Detection | 924 | [27] |
| FCV-R |  | CAGTGACAATACACCCAGAA |  |  |  |  |
| FKoV-F | RT-PCR | CATGCTCCTCGGTGGTCTCA | 7357-7968 | Detection | 631 | [28] |
| FKoV-R |  | GTCCGGGTCCATCACAGGGT |  |  |  |  |
| FNoV-F | RT-PCR | GCCCACTGGATWTACACCCTCTC | 4655-4993 | Detection | 338 | [29] |
| FNoV-R |  | CTGATGGTTGGGTCCTCTGGTCCA |  |  |  |  |
| FAstV-F | RT-PCR | GAAATGGATTGGACACGYTAYGA | 3541-3959 | Detection | 419 | T/CVMA 47 |
| FAstV-R |  | GGCTTGACCCACATRCCGAA |  |  |  |  |
| FRV-F | RT-PCR | ACCATCTACACATGACCCTC | 963-1049 | Detection | 87 | [30] |
| FRV-R |  | GGTCACATAACGCCCC |  |  |  |  |
| FPV-VP2-F | PCR | ATGAGTGATGGAGCAGTTCAA |  | VP2 sequencing | 1755 | This study |
| FPV-VP2-R |  | TTAATATAATTTTCTAGGTGC |  |  |  |  |
