# Supplementary material for: Prevalence and Molecular Evolution of Parvovirus in Cats in Eastern Shandong, China, between 2021 and 2022
Source: Transbound Emerg Dis. 2024 Jan 5;2024:5514806. doi: 10.1155/2024/5514806 (PMC12016963; doi:10.1155/2024/5514806)
Supplement: Supplementary 2 — Key amino-acid residues in VP2 protein of 21 parvoviruses and reference FPV/CPV-2 strains from GenBank. [file 5514806.f2.zip › Table S2a.pdf]

718 complete VP2 sequences of FPV used in Figure 1.

Genbank No./Sample	collection country	collection date	host	Genotype/Group	80	87	91	93	103	232	297	300	305	323	324	370	426	564	568
OQ535496/SDQD-6	China	2022		FPV	K	M	S	K	V	V	S	A	D	D	Y	Q	N	N	A
OQ535497/SDQD-8	China	2022		FPV	K	M	S	K	V	V	S	A	D	D	Y	Q	N	N	A
OQ535498/SDQD-9	China	2022		FPV	K	M	S	K	V	V	S	A	D	D	Y	Q	N	N	A
OQ535499/SDQD-12	China	2022		FPV	K	M	S	K	V	V	S	A	D	D	Y	Q	N	N	A
OQ535500/SDQD-13	China	2022		FPV	K	M	S	K	V	V	S	A	D	D	Y	Q	N	N	A
OQ535501/SDQD-14	China	2022		FPV	K	M	S	K	V	V	S	A	D	D	Y	Q	N	N	A
OQ535502/SDQD-15	China	2022		FPV	K	M	S	K	V	V	S	A	D	D	Y	Q	N	N	A
OQ535503/SDQD-19	China	2022		FPV	K	M	S	K	V	V	S	A	D	D	Y	Q	N	N	A
OQ535504/SDQD-21	China	2022		FPV	K	M	S	K	V	V	S	A	D	D	Y	Q	N	N	A
OQ535505/SDQD-23	China	2022		FPV	K	M	S	K	V	V	S	A	D	D	Y	Q	N	N	A
OQ535506/SDQD-24	China	2022		FPV	K	M	S	K	V	V	S	A	D	D	Y	Q	N	N	A
OQ535495/SDYT1	China	2022		FPV	K	M	A	K	V	V	S	A	D	D	Y	Q	N	N	A
OQ535507/SDYT22	China	2022		FPV	K	M	A	K	V	V	S	A	D	D	Y	Q	N	N	A
OQ535508/SDYT27	China	2022		FPV	K	M	S	K	V	V	S	A	D	D	Y	Q	N	N	A
OQ535509/SDYT28	China	2022		FPV	K	M	S	K	V	V	S	A	D	D	Y	Q	N	N	A
OQ535510/SDYT29	China	2022		FPV	K	M	S	K	V	V	S	A	D	D	Y	Q	N	N	A
OQ535511/SDYT30	China	2022		FPV	K	M	S	K	V	V	S	A	D	D	Y	Q	N	N	A
OQ535512/SDYT39	China	2021		FPV	K	M	S	K	V	V	S	A	D	D	Y	Q	N	N	A
OQ535513/SDYT40	China	2021		FPV	K	M	S	K	V	V	S	A	D	D	Y	Q	N	N	A
OQ535514/SDYT41	China	2021		FPV	K	M	S	K	V	V	S	A	D	D	Y	Q	N	N	A
EU018142.1	Argentina	2007		FPV-G1	K	M	S	K	V	I	S	A	D	D	Y	Q	N	N	A
EU018143.1	Argentina	2007		FPV-G1	K	M	S	K	V	I	S	A	D	D	Y	Q	N	N	A
EU018144.1	Argentina	2007		FPV-G1	K	M	S	K	V	I	S	A	D	D	Y	Q	N	N	A
EU018145.1	Argentina	2007		FPV-G1	K	M	S	K	V	I	S	A	D	D	Y	Q	N	N	A
EU145593.1	Hungary	2007		FPV-G1	K	M	A	K	V	V	S	A	D	D	Y	Q	N	S	A
EU252146.1	South Korea	2007		FPV-G1	K	M	S	K	V	V	S	A	D	D	Y	Q	N	N	A
EU252147.1	South Korea	2007		FPV-G1	K	M	S	K	V	V	S	A	D	D	Y	Q	N	N	A
EU360958.1	Hungary	2007		FPV-G1	K	M	S	K	V	V	S	A	D	D	Y	Q	N	N	A
EU360959.1	Hungary	2007		FPV-G1	K	M	S	K	V	V	S	A	D	D	Y	Q	N	S	G
EU498699.1	Italy	2006		FPV-G1	K	M	S	K	V	V	S	A	D	D	Y	Q	N	N	A
EU498706.1	Italy	2006		FPV-G1	K	M	S	K	V	V	S	A	D	D	Y	Q	N	N	A
FJ440711.1	Argentina	2007		FPV-G1	K	M	S	K	V	I	S	A	D	D	Y	Q	N	N	A
FJ440712.1	Argentina	2007		FPV-G1	K	M	S	K	V	I	S	A	D	D	Y	Q	N	N	A
FJ440713.1	Argentina	2007		FPV-G1	K	M	S	K	V	I	S	A	D	D	Y	Q	N	N	A
FJ440714.1	Argentina	2007		FPV-G1	K	M	S	K	V	I	S	A	D	D	Y	Q	N	N	A
HQ184195.1	South Korea	2008		FPV-G1	K	M	S	K	V	V	S	A	D	D	Y	Q	N	N	A
MW847204.1	Italy	2019		FPV-G1	K	M	S	K	V	V	S	A	D	D	Y	Q	N	N	A
MW847205.1	Italy	2019		FPV-G1	K	M	S	K	V	V	S	A	D	D	Y	Q	N	N	A
AB000050.1	Japan	2009		FPV-G2-1	K	M	A	K	V	V	S	A	D	D	Y	Q	N	N	A
AB000052.1	Japan	1996		FPV-G2-1	K	M	A	K	V	V	S	A	D	D	Y	Q	N	N	A
AB000054.1	Japan	1996		FPV-G2-1	K	M	A	K	V	V	S	A	D	D	Y	Q	N	N	A
AB000059.1	Japan	1996		FPV-G2-1	K	M	A	K	V	V	S	A	D	D	Y	Q	N	N	A
AB000061.1	Japan	1996		FPV-G2-1	K	M	A	K	V	V	S	A	D	D	Y	Q	N	N	A
AB000064.1	Japan	1996		FPV-G2-1	K	M	A	K	V	V	S	A	D	D	Y	Q	N	N	A
AB000066.1	Japan	1996		FPV-G2-1	K	M	A	K	V	V	S	A	D	D	Y	Q	N	N	A
AB000068.1	Japan	1996		FPV-G2-1	K	M	A	K	V	V	S	A	D	D	Y	Q	N	N	A
AB000070.1	Japan	1996		FPV-G2-1	K	M	A	K	V	I	S	A	D	D	Y	Q	N	N	A
AB262659.1	Japan	2006		FPV-G2-1	K	M	A	K	V	V	S	A	D	D	Y	Q	N	N	A
D88286.1	Japan	1996		FPV-G2-1	K	M	A	K	V	V	S	A	D	D	Y	Q	N	N	A
EU659111.1	USA	1967		FPV-G2-1	K	M	A	K	V	V	S	A	D	D	Y	Q	N	N	A
KJ813893.1	USA	2013		FPV-G2-1	K	M	A	K	V	V	S	A	D	D	Y	Q	N	N	A
KT240134.1	Portugal	2013		FPV-G2-1	K	M	A	K	V	V	S	A	D	D	Y	Q	N	N	A
KT240136.1	Portugal	2014		FPV-G2-1	K	M	A	K	V	V	S	A	D	D	Y	Q	N	N	A
M24004.1	USA	1988		FPV-G2-1	K	M	A	K	V	I	S	A	D	D	Y	Q	N	N	A
M38246.1 (vaccine)	USA	1996		FPV-G2-1	K	M	A	K	V	V	S	A	D	D	Y	Q	N	N	A
MK570637.1	Australia	2015		FPV-G2-1	K	M	A	K	V	V	S	A	D	D	Y	Q	N	N	A
MK570638.1	Australia	2015		FPV-G2-1	K	M	A	K	V	V	S	A	D	D	Y	Q	N	N	A
MK570639.1	Australia	2015		FPV-G2-1	K	M	A	K	V	V	S	A	D	D	Y	Q	N	N	A
MK570640.1	Australia	2015		FPV-G2-1	K	M	A	K	V	V	S	A	D	D	Y	Q	N	N	A
MK570641.1	Australia	2015		FPV-G2-1	K	M	A	K	V	V	S	A	D	D	Y	Q	N	N	A
MK570642.1	Australia	2015		FPV-G2-1	K	M	A	K	V	V	S	A	D	D	Y	Q	N	N	A
MK570643.1	Australia	2015		FPV-G2-1	K	M	A	K	V	V	S	A	D	D	Y	Q	N	N	A
MK570646.1	Australia	2015		FPV-G2-1	K	M	A	K	V	V	S	A	D	D	Y	Q	N	N	A
MK570647.1	Australia	2015		FPV-G2-1	K	M	A	K	V	V	S	A	D	D	Y	Q	N	N	A
MK570648.1	Australia	2016		FPV-G2-1	K	M	A	K	V	V	S	A	D	D	Y	Q	N	N	A
MK570649.1	Australia	2016		FPV-G2-1	K	M	A	K	V	V	S	A	D	D	Y	Q	N	N	A
MK570650.1	Australia	2016		FPV-G2-1	K	M	A	K	V	V	S	A	D	D	Y	Q	N	N	A
MK570651.1	Australia	2016		FPV-G2-1	K	M	A	K	V	V	S	A	D	D	Y	Q	N	N	A
MK570652.1	Australia	2016		FPV-G2-1	K	M	A	K	V	V	S	A	D	D	Y	Q	N	N	A
MK570653.1	Australia	2016		FPV-G2-1	K	M	A	K	V	V	S	A	D	D	Y	Q	N	N	A
MK570695.1	Australia	2017		FPV-G2-1	K	M	A	K	V	V	S	A	D	D	Y	Q	N	N	A
MK570749.1	Australia	2016		FPV-G2-1	K	M	A	K	V	V	S	A	D	D	Y	Q	N	N	A
MT078767.1	India	2019		FPV-G2-1	K	M	A	K	V	V	S	A	D	D	Y	Q	N	N	A
MT078768.1	India	2019		FPV-G2-1	K	M	A	K	V	V	S	A	D	D	Y	Q	N	N	A
MT078769.1	India	2019		FPV-G2-1	K	M	A	K	V	V	S	A	D	D	Y	Q	N	N	A
MT078770.1	India	2019		FPV-G2-1	K	M	A	K	V	V	S	A	D	D	Y	Q	N	N	A
MT078771.1	India	2019		FPV-G2-1	K	M	A	K	V	V	S	A	D	D	Y	Q	N	N	A
MZ362883.1	Australia	2019	canine	FPV-G2-1	K	M	A	K	V	V	S	A	D	D	Y	Q	N	N	A
AB000056.1	Japan	1996		FPV-G2-2	K	M	A	K	V	V	S	A	D	D	Y	Q	N	N	A
D78584.1	Japan	1995		FPV-G2-2	K	M	A	K	V	V	S	A	D	D	Y	Q	N	N	A
D88287.1	Japan	1996		FPV-G2-2	K	M	A	K	V	I	S	A	D	D	Y	Q	N	N	A
EU252145.1	South Korea	2007		FPV-G2-2	K	M	A	K	V	V	S	A	D	D	Y	Q	N	N	A
EU498680.1	Italy	2008		FPV-G2-2	K	M	A	K	V	I	S	A	D	D	Y	Q	N	N	A
EU498681.1	Italy	2008		FPV-G2-2	K	M	A	K	V	I	S	A	D	D	Y	Q	N	N	A
EU498701.1	Italy	2006		FPV-G2-2	K	M	A	K	V	V	S	A	D	D	Y	Q	N	N	A
EU498707.1	Italy	2006		FPV-G2-2	K	M	A	K	V	V	S	A	D	D	Y	Q	N	N	A
EU498713.1	United Kingdom	2006		FPV-G2-2	K	M	A	K	V	V	S	A	D	D	Y	Q	N	N	A
EU498714.1	United Kingdom	2006		FPV-G2-2	K	M	A	K	V	I	S	A	D	D	Y	Q	N	N	A
EU498717.1	United Kingdom	2007		FPV-G2-2	K	M	A	K	V	I	S	A	D	D	Y	Q	N	N	A
EU498718.1	Italy	2007		FPV-G2-2	K	M	A	K	V	V	S	A	D	D	Y	Q	N	N	A

EU498719.1	United Kingdom	2007		FPV-G2-2	K	M	A	K	V	I	S	A	D	D	Y	Q	N	N	A
EU498720.1	Italy	2007		FPV-G2-2	K	M	A	K	V	V	S	A	D	D	Y	Q	N	N	A
EU659112.1	USA	1964		FPV-G2-2	K	M	A	K	V	V	S	A	D	D	Y	Q	N	N	A
HQ184192.1	South Korea	2008		FPV-G2-2	K	M	A	K	V	V	S	A	D	D	Y	Q	N	N	A
HQ184193.1	South Korea	2008		FPV-G2-2	K	M	A	K	V	V	S	A	D	D	Y	Q	N	N	A
HQ184197.1	South Korea	2008		FPV-G2-2	K	M	A	K	V	V	S	A	D	D	Y	Q	N	N	A
M24002.1 1	USA	1989		FPV-G2-2	K	M	A	K	V	I	S	A	D	D	Y	Q	N	N	A
MK570676.1	Australia	2018		FPV-G2-2	K	M	A	K	V	I	S	A	D	D	Y	Q	N	N	A
MK570696.1	New Zealand	2017		FPV-G2-2	K	M	A	K	V	I	S	A	D	D	Y	Q	N	N	A
MK570697.1	New Zealand	2017		FPV-G2-2	K	M	A	K	V	I	S	A	D	D	Y	Q	N	N	A
MK570698.1	New Zealand	2017		FPV-G2-2	K	M	A	K	V	I	S	A	D	D	Y	Q	N	N	A
MK570699.1	New Zealand	2017		FPV-G2-2	K	M	A	K	V	I	S	A	D	D	Y	Q	N	N	A
MK570700.1	New Zealand	2017		FPV-G2-2	K	M	A	K	V	I	S	A	D	D	Y	Q	N	N	A
MK570701.1	New Zealand	2017		FPV-G2-2	K	M	A	K	V	I	S	A	D	D	Y	Q	N	N	A
MK570702.1	New Zealand	2017		FPV-G2-2	K	M	A	K	V	I	S	A	D	D	Y	Q	N	N	A
MK570703.1	New Zealand	2017		FPV-G2-2	K	M	A	K	V	I	S	A	D	D	Y	Q	N	N	A
MK570704.1	New Zealand	2017		FPV-G2-2	K	M	A	K	V	I	S	A	D	D	Y	Q	N	N	A
MK570707.1	Australia	2018		FPV-G2-2	K	M	A	K	V	I	S	A	D	D	Y	Q	N	N	A
MK570709.1	Australia	2017		FPV-G2-2	K	M	A	K	V	I	S	A	D	D	Y	Q	N	N	A
MK570714.1	Australia	2018		FPV-G2-2	K	M	A	K	V	I	S	A	D	D	Y	Q	N	N	A
MK570715.1	Australia	2018		FPV-G2-2	K	M	A	K	V	I	S	A	D	D	Y	Q	N	N	A
MK570737.1	Australia	2017		FPV-G2-2	K	M	A	K	V	I	S	A	D	D	Y	Q	N	N	A
MN400979.1	South Korea	2017		FPV-G2-2	K	M	A	K	V	V	S	A	D	D	Y	Q	N	N	A
MN603976.1	Australia	2010		FPV-G2-2	K	M	A	K	V	V	S	A	D	D	Y	Q	N	N	A
MW847159.1	Italy	2012		FPV-G2-2	K	M	A	K	V	I	S	A	D	D	Y	Q	N	N	A
MW847201.1	Italy	2018		FPV-G2-2	K	M	A	K	V	I	S	A	D	D	Y	Q	N	N	A
MW926314.1	United Kingdom	2019		FPV-G2-2	K	M	A	K	V	I	S	A	D	D	Y	Q	N	N	A
OM640096.1	Canada	2018		FPV-G2-2	K	M	A	K	V	I	S	A	D	D	Y	Q	N	N	A
ON605652.1	Australia	2022		FPV-G2-2	K	M	A	K	V	I	S	A	D	D	Y	Q	N	N	A
X55115.1	Australia	1990		FPV-G2-2	K	M	A	K	V	V	S	A	D	D	Y	Q	N	N	A
AB054225.1	Japan	2001		FPV-G3A	K	M	A	N	A	V	S	A	D	N	Y	Q	N	N	A
AB054226.1	Japan	2000		FPV-G3A	K	M	A	K	V	V	S	A	D	D	Y	Q	N	N	A
AB054227.1	Japan	2001		FPV-G3A	K	M	A	K	V	V	S	A	D	D	Y	Q	N	N	A
AY606131.1	France	2004		FPV-G3A	K	M	A	K	V	V	S	A	D	D	Y	Q	N	N	A
EU221281.1	Portugal	2006		FPV-G3A	K	M	A	K	V	V	S	A	D	D	Y	Q	N	N	A
HQ184189.1	South Korea	2009		FPV-G3A	K	M	A	K	V	V	S	A	D	D	Y	Q	N	N	A
HQ184190.1	South Korea	2008		FPV-G3A	K	M	A	K	V	V	S	A	D	D	Y	Q	N	N	A
HQ184191.1	South Korea	2008		FPV-G3A	K	M	A	K	V	V	S	A	D	D	Y	Q	N	N	A
HQ184194.1	South Korea	2008		FPV-G3A	K	M	A	K	V	V	S	A	D	D	Y	Q	N	N	A
HQ184196.1	South Korea	2008		FPV-G3A	Q	M	A	K	V	V	S	A	D	D	Y	Q	N	N	A
HQ184198.1	South Korea	2008		FPV-G3A	K	M	A	K	V	V	S	A	D	D	H	Q	N	N	A
HQ184199.1	South Korea	2008		FPV-G3A	K	M	A	K	V	V	S	A	D	D	C	Q	N	N	A
HQ184201.1	South Korea	2008		FPV-G3A	K	M	A	K	V	V	S	A	N	D	Y	Q	N	N	A
HQ184202.1	South Korea	2008		FPV-G3A	K	M	A	K	V	I	S	A	D	D	Y	Q	N	N	A
HQ184203.1	South Korea	2008		FPV-G3A	K	M	A	K	V	V	S	A	D	D	Y	Q	N	N	A
HQ184204.1	South Korea	2008		FPV-G3A	K	M	A	K	V	I	S	A	D	D	Y	Q	N	N	A
KT240130.1	Portugal	2008		FPV-G3A	K	M	A	K	V	V	S	A	D	D	Y	Q	N	N	A
MK357738.1	Viet Nam	2017	canine	FPV-G3A	K	M	A	K	V	V	S	A	D	D	Y	Q	N	N	A
MK357739.1	Viet Nam	2017	canine	FPV-G3A	K	M	A	K	V	V	S	A	D	D	Y	Q	N	N	A
MK357740.1	Viet Nam	2017	canine	FPV-G3A	K	M	A	K	V	V	S	A	D	D	Y	Q	N	N	A
MK357741.1	Viet Nam	2018	canine	FPV-G3A	K	M	A	K	V	V	S	A	D	D	Y	Q	N	N	A
MK357742.1	Viet Nam	2018	canine	FPV-G3A	K	M	A	K	V	V	S	A	D	D	Y	Q	N	N	A
MK357743.1	Viet Nam	2018	canine	FPV-G3A	K	M	A	K	V	V	S	A	D	D	Y	Q	N	N	A
MT857268.1	Viet Nam	2018		FPV-G3A	K	M	A	K	V	V	S	A	D	D	Y	Q	N	N	A
MT857269.1	Viet Nam	2018		FPV-G3A	K	M	A	K	V	V	S	A	D	D	Y	Q	N	N	A
MT857270.1	Viet Nam	2018		FPV-G3A	K	M	A	K	V	V	S	A	D	D	Y	Q	N	N	A
MT857271.1	Viet Nam	2018		FPV-G3A	K	M	A	K	V	V	S	A	D	D	Y	Q	N	N	A
MT857272.1	Viet Nam	2018		FPV-G3A	K	M	A	K	V	V	S	A	D	D	Y	Q	N	N	A
MT857273.1	Viet Nam	2019		FPV-G3A	K	M	A	K	V	V	S	A	D	D	Y	Q	N	N	A
MT857274.1	Viet Nam	2019		FPV-G3A	K	M	A	K	V	V	S	A	D	D	Y	Q	N	N	A
MT857275.1	Viet Nam	2019		FPV-G3A	K	M	A	K	V	V	S	A	D	D	Y	Q	N	N	A
MT857276.1	Viet Nam	2019		FPV-G3A	K	M	A	K	V	V	S	A	D	D	Y	Q	N	N	A
MT857277.1	Viet Nam	2019		FPV-G3A	K	M	A	K	V	V	S	A	D	D	Y	Q	N	N	A
MT857278.1	Viet Nam	2019		FPV-G3A	K	M	A	K	V	V	S	A	D	D	Y	Q	N	N	A
MT857279.1	Viet Nam	2019		FPV-G3A	K	M	A	K	V	V	S	A	D	D	Y	Q	N	N	A
MT857280.1	Viet Nam	2019		FPV-G3A	K	M	A	K	V	V	S	A	D	D	Y	Q	N	N	A
MT857281.1	Viet Nam	2019		FPV-G3A	K	M	A	K	V	V	S	A	D	D	Y	Q	N	N	A
MT857282.1	Viet Nam	2019		FPV-G3A	K	M	A	K	V	V	S	A	D	D	Y	Q	N	N	A
MT857283.1	Viet Nam	2018		FPV-G3A	K	M	A	K	V	V	S	A	D	D	Y	Q	N	N	A
MT857284.1	Viet Nam	2018		FPV-G3A	K	M	A	K	V	V	S	A	D	D	Y	Q	N	N	A
MT857285.1	Viet Nam	2018		FPV-G3A	K	M	A	K	V	V	S	A	D	D	Y	Q	N	N	A
MT857286.1	Viet Nam	2018		FPV-G3A	K	M	A	K	V	V	S	A	D	D	Y	Q	N	N	A
MW847185.1	Italy	2017		FPV-G3A	K	M	A	K	V	V	S	A	D	D	Y	Q	N	N	A
MW847196.1	Italy	2018		FPV-G3A	K	M	A	K	V	V	S	A	D	D	Y	Q	N	N	A
MW847197.1	Italy	2018		FPV-G3A	K	M	A	K	V	V	S	A	D	D	Y	Q	N	N	A
MW847202.1	Italy	2018		FPV-G3A	K	M	A	K	V	V	S	A	D	D	Y	Q	N	N	A
MW847206.1	Italy	2019		FPV-G3A	K	M	A	K	V	V	S	A	D	D	Y	Q	N	N	A
MW847207.1	Italy	2019		FPV-G3A	K	M	A	K	V	V	S	A	D	D	Y	Q	N	N	A
MW847209.1	Italy	2019		FPV-G3A	K	M	A	K	V	V	S	A	D	D	Y	Q	N	N	A
MZ391097.1	Turkey	2020		FPV-G3A	K	M	A	K	V	V	S	A	D	D	Y	Q	N	N	A
OM638042.1	Italy	2021	canine	FPV-G3A	K	M	A	K	V	V	S	A	D	D	Y	Q	N	N	A
OP153929.1	South Korea	2019		FPV-G3A	K	M	A	K	V	V	S	A	D	D	Y	Q	N	N	A
EU498693.1	Italy	2004		FPV-G3B	K	M	A	K	V	V	S	A	D	D	Y	Q	N	N	A
EU498698.1	Italy	2006		FPV-G3B	K	M	A	K	V	V	S	A	D	D	Y	Q	N	N	A
KX943318.1	Italy	2015		FPV-G3B	K	M	A	K	V	V	S	A	D	D	Y	Q	N	N	A
MZ391095.1	Turkey	2020		FPV-G3B	K	M	A	K	V	V	S	A	D	D	Y	Q	N	N	A
MK570654.1	Australia	2017		FPV-G3C-1	K	M	A	K	V	I	S	A	D	D	Y	Q	N	N	A
MK570655.1	Australia	2017		FPV-G3C-1	K	M	A	K	V	I	S	A	D	D	Y	Q	N	N	A
MK570656.1	Australia	2017		FPV-G3C-1	K	M	A	K	V	I	S	A	D	D	Y	Q	N	N	A
MK570657.1	Australia	2017		FPV-G3C-1	K	M	A	K	V	I	S	A	D	D	Y	Q	N	N	A
MK570658.1	Australia	2017		FPV-G3C-1	K	M	A	K	V	I	S	A	D	D	Y	Q	N	N	A
MK570659.1	Australia	2017		FPV-G3C-1	K	M	A	K	V	I	S	A	D	D	Y	Q	N	N	A

MK570660.1	Australia	2017		FPV-G3C-1	K	M	A	K	V	I	S	A	D	D	Y	Q	N	N	A
MK570661.1	Australia	2017		FPV-G3C-1	K	M	A	K	V	I	S	A	D	D	Y	Q	N	N	A
MK570662.1	Australia	2017		FPV-G3C-1	K	M	A	K	V	I	S	A	D	D	Y	Q	N	N	A
MK570663.1	Australia	2017		FPV-G3C-1	K	M	A	K	V	V	S	A	D	D	Y	Q	N	N	A
MK570664.1	Australia	2017		FPV-G3C-1	K	M	A	K	V	V	S	A	D	D	Y	Q	N	N	A
MK570665.1	Australia	2017		FPV-G3C-1	K	M	A	K	V	I	S	A	D	D	Y	Q	N	N	A
MK570666.1	Australia	2017		FPV-G3C-1	K	M	A	K	V	I	S	A	D	D	Y	Q	N	N	A
MK570667.1	Australia	2017		FPV-G3C-1	K	M	A	K	V	V	S	A	D	D	Y	Q	N	N	A
MK570668.1	Australia	2017		FPV-G3C-1	K	M	A	K	V	V	S	A	D	D	Y	Q	N	N	A
MK570669.1	Australia	2017		FPV-G3C-1	K	M	A	K	V	V	S	A	D	D	Y	Q	N	N	A
MK570677.1	Australia	2017		FPV-G3C-1	K	M	A	K	V	V	S	A	D	D	Y	Q	N	N	A
MK570678.1	Australia	2017		FPV-G3C-1	K	M	A	K	V	V	S	A	D	D	Y	Q	N	N	A
MK570679.1	Australia	2017		FPV-G3C-1	K	M	A	K	V	V	S	A	D	D	Y	Q	N	N	A
MK570670.1	Australia	2017		FPV-G3C-1	K	M	A	K	V	I	S	A	D	D	Y	Q	N	N	A
MK570671.1	Australia	2017		FPV-G3C-1	K	M	A	K	V	I	S	A	D	D	Y	Q	N	N	A
MK570672.1	Australia	2017		FPV-G3C-1	K	M	A	K	V	I	S	A	D	D	Y	Q	N	N	A
MK570673.1	Australia	2017		FPV-G3C-1	K	M	A	K	V	I	S	A	D	D	Y	Q	N	N	A
MK570674.1	Australia	2017		FPV-G3C-1	K	M	A	K	V	I	S	A	D	D	Y	Q	N	N	A
MK570680.1	Australia	2017		FPV-G3C-1	K	M	A	K	V	I	S	A	D	D	Y	Q	N	N	A
MK570681.1	Australia	2017		FPV-G3C-1	K	M	A	K	V	I	S	A	D	D	Y	Q	N	N	A
MK570682.1	Australia	2017		FPV-G3C-1	K	M	A	K	V	I	S	A	D	D	Y	Q	N	N	A
MK570683.1	Australia	2017		FPV-G3C-1	K	M	A	K	V	I	S	A	D	D	Y	Q	N	N	A
MK570684.1	Australia	2017		FPV-G3C-1	K	M	A	K	V	V	S	A	D	D	Y	Q	N	N	A
MK570685.1	Australia	2017		FPV-G3C-1	K	M	A	K	V	V	S	A	D	D	Y	Q	N	N	A
MK570686.1	Australia	2017		FPV-G3C-1	K	M	A	K	V	V	S	A	D	D	Y	Q	N	N	A
MK570687.1	Australia	2017		FPV-G3C-1	K	M	A	K	V	V	S	A	D	D	Y	Q	N	N	A
MK570688.1	Australia	2017		FPV-G3C-1	K	M	A	K	V	V	S	A	D	D	Y	Q	N	N	A
MK570689.1	Australia	2017		FPV-G3C-1	K	M	A	K	V	V	S	A	D	D	Y	Q	N	N	A
MK570690.1	Australia	2017		FPV-G3C-1	K	M	A	K	V	V	S	A	D	D	Y	Q	N	N	A
MK570691.1	Australia	2017		FPV-G3C-1	K	M	A	K	V	V	S	A	D	D	Y	Q	N	N	A
MK570692.1	Australia	2017		FPV-G3C-1	K	M	A	K	V	I	S	A	D	D	Y	Q	N	N	A
MK570693.1	Australia	2017		FPV-G3C-1	K	M	A	K	V	V	S	A	D	D	Y	Q	N	N	A
MK570694.1	Australia	2017		FPV-G3C-1	K	M	A	K	V	V	S	A	D	D	Y	Q	N	N	A
MK570705.1	Australia	2017		FPV-G3C-1	K	M	A	K	V	I	S	A	D	D	Y	Q	N	N	A
MK570706.1	Australia	2018		FPV-G3C-1	K	M	A	K	V	I	S	A	D	D	Y	Q	N	N	A
MK570708.1	Australia	2017		FPV-G3C-1	K	M	A	K	V	I	S	A	D	D	Y	Q	N	N	A
MK570710.1	Australia	2017		FPV-G3C-1	K	M	A	K	V	I	S	A	D	D	Y	Q	N	N	A
MK570711.1	Australia	2018		FPV-G3C-1	K	M	A	K	V	V	S	A	D	D	Y	Q	N	N	A
MK570712.1	Australia	2018		FPV-G3C-1	K	M	A	K	V	V	S	A	D	D	Y	Q	N	N	A
MK570713.1	Australia	2018		FPV-G3C-1	K	M	A	K	V	V	S	A	D	D	Y	Q	N	N	A
MK570725.1	United Arab Emirate	2017		FPV-G3C-1	K	M	A	K	V	V	S	A	D	D	Y	Q	N	N	A
MK570726.1	Australia	2017		FPV-G3C-1	K	M	A	K	V	V	S	A	D	D	Y	Q	N	N	A
MK570727.1	Australia	2017		FPV-G3C-1	K	M	A	K	V	I	S	A	D	D	Y	Q	N	N	A
MK570728.1	Australia	2016		FPV-G3C-1	K	M	A	K	V	I	S	A	D	D	Y	Q	N	N	A
MK570729.1	Australia	2017		FPV-G3C-1	K	M	A	K	V	I	S	A	D	D	Y	Q	N	N	A
MK570730.1	Australia	2017		FPV-G3C-1	K	M	A	K	V	V	S	A	D	D	Y	Q	N	N	A
MK570731.1	Australia	2017		FPV-G3C-1	K	M	A	K	V	V	S	A	D	D	Y	Q	N	N	A
MK570732.1	Australia	2017		FPV-G3C-1	K	M	A	K	V	V	S	A	D	D	Y	Q	N	N	A
MK570733.1	Australia	2017		FPV-G3C-1	K	M	A	K	V	I	S	A	D	D	Y	Q	N	N	A
MK570734.1	Australia	2017		FPV-G3C-1	K	M	A	K	V	V	S	A	D	D	Y	Q	N	N	A
MK570735.1	Australia	2017		FPV-G3C-1	K	M	A	K	V	V	S	A	D	D	Y	Q	N	N	A
MK570736.1	Australia	2017		FPV-G3C-1	K	M	A	K	V	V	S	A	D	D	Y	Q	N	N	A
MK570738.1	Australia	2017		FPV-G3C-1	K	M	A	K	V	I	S	A	D	D	Y	Q	N	N	A
MK570739.1	Australia	2017		FPV-G3C-1	K	M	A	K	V	I	S	A	D	D	Y	Q	N	N	A
MK570740.1	Australia	2016		FPV-G3C-1	K	M	A	K	V	I	S	A	D	D	Y	Q	N	N	A
MK570741.1	Australia	2017		FPV-G3C-1	K	M	A	K	V	V	S	A	D	D	Y	Q	N	N	A
MK570742.1	Australia	2017		FPV-G3C-1	K	M	A	K	V	V	S	A	D	D	Y	Q	N	N	A
MK570743.1	Australia	2017		FPV-G3C-1	K	M	A	K	V	V	S	A	D	D	Y	Q	N	N	A
MK570744.1	Australia	2017		FPV-G3C-1	K	M	A	K	V	V	S	A	D	D	Y	Q	N	N	A
MK570745.1	Australia	2017		FPV-G3C-1	K	M	A	K	V	V	S	A	D	D	Y	Q	N	N	A
MK570746.1	Australia	2017		FPV-G3C-1	K	M	A	K	V	V	S	A	D	D	Y	Q	N	N	A
MK570747.1	Australia	2017		FPV-G3C-1	K	M	A	K	V	V	S	A	D	D	Y	Q	N	N	A
MK570748.1	Australia	2017		FPV-G3C-1	K	M	A	K	V	V	S	A	D	D	Y	Q	N	N	A
MW035310.1	South Korea	2018	canine	FPV-G3C-1	K	M	A	K	V	V	S	A	D	D	Y	Q	N	N	A
MW589473.1	Thailand	2020		FPV-G3C-1	K	M	A	K	V	V	S	A	D	D	Y	Q	N	N	A
MZ742163.1	Australia	2017		FPV-G3C-1	K	M	A	K	V	I	S	A	D	D	Y	Q	N	N	A
MZ742164.1	Australia	2017		FPV-G3C-1	K	M	A	K	V	V	S	A	D	D	Y	Q	N	N	A
MZ742165.1	Australia	2017		FPV-G3C-1	K	M	A	K	V	V	S	A	D	D	Y	Q	N	N	A
MZ742166.1	Australia	2017		FPV-G3C-1	K	M	A	K	V	V	S	A	D	D	Y	Q	N	N	A
MZ742167.1	Australia	2017		FPV-G3C-1	K	M	A	K	V	I	S	A	D	D	Y	Q	N	N	A
MZ742168.1	Australia	2017		FPV-G3C-1	K	M	A	K	V	V	S	A	D	D	Y	Q	N	N	A
MZ742169.1	Australia	2017		FPV-G3C-1	K	M	A	K	V	I	S	A	D	D	Y	Q	N	N	A
MZ742170.1	Australia	2017		FPV-G3C-1	K	M	A	K	V	V	S	A	D	D	Y	Q	N	N	A
MZ742171.1	Australia	2017		FPV-G3C-1	K	M	A	K	V	V	S	A	D	D	Y	Q	N	N	A
MZ742172.1	Australia	2017		FPV-G3C-1	K	M	A	K	V	V	S	A	D	D	Y	Q	N	N	A
MZ742173.1	Australia	2017		FPV-G3C-1	K	M	A	K	V	I	S	A	D	D	Y	Q	N	N	A
MZ742174.1	Australia	2017		FPV-G3C-1	K	M	A	K	V	I	S	A	D	D	Y	Q	N	N	A
MZ742175.1	Australia	2017		FPV-G3C-1	K	M	A	K	V	V	S	A	D	D	Y	Q	N	N	A
MZ742176.1	Australia	2017		FPV-G3C-1	K	M	A	K	V	V	S	A	D	D	Y	Q	N	N	A
MZ742177.1	Australia	2017		FPV-G3C-1	K	M	A	K	V	V	S	A	D	D	Y	Q	N	N	A
MZ742178.1	Australia	2017		FPV-G3C-1	K	M	A	K	V	V	S	A	D	D	Y	Q	N	N	A
MZ742179.1	Australia	2017		FPV-G3C-1	K	M	A	K	V	V	S	A	D	D	Y	Q	N	N	A
MZ742180.1	Australia	2017		FPV-G3C-1	K	M	A	K	V	V	S	A	D	D	Y	Q	N	N	A
EU221278.1	Portugal	2005		FPV-G3C-2	K	M	A	K	V	V	S	A	D	D	Y	Q	N	N	A
EU221279.1	Portugal	2005		FPV-G3C-2	K	M	A	K	V	V	S	A	D	D	Y	Q	N	N	A
EU221280.1	Portugal	2006		FPV-G3C-2	K	M	A	K	V	V	S	A	D	D	Y	Q	N	N	A
EU498684.1	Italy	2002		FPV-G3C-2	K	M	A	K	V	V	S	A	D	D	Y	Q	N	N	A
EU498685.1	Italy	2003		FPV-G3C-2	K	M	A	K	V	V	S	A	D	D	Y	Q	N	N	A
EU498688.1	Italy	2004		FPV-G3C-2	K	M	A	K	V	V	S	A	D	D	Y	Q	N	N	A
EU498689.1	Italy	2004		FPV-G3C-2	K	M	A	K	V	V	S	A	D	D	Y	Q	N	N	A
EU498691.1	Italy	2004		FPV-G3C-2	K	M	A	K	V	V	S	A	D	D	Y	Q	N	N	A
EU498694.1	Italy	2005		FPV-G3C-2	K	M	A	K	V	V	S	A	D	D	Y	Q	N	N	A

EU498695.1	Italy	2005		FPV-G3C-2	K	M	A	K	V	V	S	A	D	D	Y	Q	N	N	A
EU498696.1	Italy	2006		FPV-G3C-2	K	M	A	K	V	V	S	A	D	D	Y	Q	N	N	A
EU498697.1	Italy	2006		FPV-G3C-2	K	M	A	K	V	V	S	A	D	D	Y	Q	N	N	A
EU498700.1	Italy	2006		FPV-G3C-2	K	M	A	K	V	V	S	A	D	D	Y	Q	N	N	A
EU498702.1	Italy	2006		FPV-G3C-2	K	M	A	K	V	V	S	A	D	D	Y	Q	N	N	A
EU498703.1	Italy	2006		FPV-G3C-2	K	M	A	K	V	V	S	A	D	D	Y	Q	N	N	A
EU498708.1	Italy	2006		FPV-G3C-2	K	M	A	K	V	V	S	A	D	D	Y	Q	N	N	A
EU498709.1	Italy	2006		FPV-G3C-2	K	M	A	K	V	V	S	A	D	D	Y	Q	N	N	A
EU498710.1	Italy	2006		FPV-G3C-2	K	M	A	K	V	V	S	A	D	D	Y	Q	N	N	A
EU498711.1	Italy	2006		FPV-G3C-2	K	M	A	K	V	V	S	A	D	D	Y	Q	N	N	A
EU498712.1	Italy	2006		FPV-G3C-2	K	M	A	K	V	V	S	A	D	D	Y	Q	N	N	A
EU498715.1	Italy	2006		FPV-G3C-2	K	M	A	K	V	V	S	A	D	D	Y	Q	N	N	A
KP769859.1	Belgium	2013		FPV-G3C-2	K	M	A	K	V	V	S	A	D	D	Y	Q	N	N	A
KT240129.1	Portugal	2007		FPV-G3C-2	K	M	A	K	V	V	S	A	D	D	Y	Q	N	N	A
KT240131.1	Portugal	2008		FPV-G3C-2	K	M	A	K	V	V	S	A	D	D	Y	Q	N	N	A
KT240133.1	Portugal	2013		FPV-G3C-2	K	M	A	K	V	V	S	A	D	D	Y	Q	N	N	A
KT240135.1	Portugal	2014		FPV-G3C-2	K	M	A	K	V	V	S	A	D	D	Y	Q	N	N	A
KU248462.1	Portugal	2007		FPV-G3C-2	K	M	A	K	V	V	S	A	D	D	Y	Q	N	N	A
KU248463.1	Portugal	2008		FPV-G3C-2	K	M	A	K	V	V	S	A	D	D	Y	Q	N	N	A
KU248464.1	Portugal	2014		FPV-G3C-2	K	M	A	K	V	V	S	A	D	D	Y	Q	N	N	A
MW847156.1	Italy	2011		FPV-G3C-2	K	M	A	K	V	V	S	A	D	D	Y	Q	N	N	A
MW847157.1	Italy	2011		FPV-G3C-2	K	M	A	K	V	V	S	A	D	D	Y	Q	N	N	A
MW847160.1	Italy	2012		FPV-G3C-2	K	M	A	K	V	V	S	A	D	D	Y	Q	N	N	A
MW847161.1	Italy	2012		FPV-G3C-2	K	M	A	K	V	V	S	A	D	D	Y	Q	N	N	A
MW847162.1	Italy	2013		FPV-G3C-2	K	M	A	K	V	V	S	A	D	D	Y	Q	N	N	A
MW847163.1	Italy	2013		FPV-G3C-2	K	M	A	K	V	V	S	A	D	D	Y	Q	N	N	A
MW847164.1	Italy	2016		FPV-G3C-2	K	M	A	K	V	V	S	A	D	D	Y	Q	N	N	A
MW847166.1	Italy	2016		FPV-G3C-2	K	M	A	K	V	V	S	A	D	D	Y	Q	N	N	A
MW847168.1	Italy	2016		FPV-G3C-2	K	M	A	K	V	V	S	A	D	D	Y	Q	N	N	A
MW847170.1	Italy	2016		FPV-G3C-2	K	M	A	K	V	V	S	A	D	D	Y	Q	N	N	A
MW847171.1	Italy	2016		FPV-G3C-2	K	M	A	K	V	V	S	A	D	D	Y	Q	N	N	A
MW847172.1	Italy	2016		FPV-G3C-2	K	M	A	K	V	V	S	A	D	D	Y	Q	N	N	A
MW847182.1	Italy	2017		FPV-G3C-2	K	M	A	K	V	V	S	A	D	D	Y	Q	N	N	A
MW847184.1	Italy	2017		FPV-G3C-2	K	M	A	K	V	V	S	A	D	D	Y	Q	N	N	A
MW847186.1	Italy	2017		FPV-G3C-2	K	M	A	K	V	V	S	A	D	D	Y	Q	N	N	A
MW847195.1	Italy	2017		FPV-G3C-2	K	M	A	K	V	V	S	A	D	D	Y	Q	N	N	A
MW847198.1	Italy	2018		FPV-G3C-2	K	M	A	K	V	V	S	A	D	D	Y	Q	N	N	A
MW847203.1	Italy	2019		FPV-G3C-2	K	M	A	K	V	V	S	A	D	D	Y	Q	N	N	A
MW847208.1	Italy	2018		FPV-G3C-2	K	M	A	K	V	V	S	A	D	D	Y	Q	N	N	A
AY665655.1	Russia	2004		FPV-G3C-2	K	M	A	K	V	V	S	A	D	D	Y	Q	N	N	A
EU498683.1	Italy	2008		FPV-G3C-2	K	M	A	K	V	V	S	A	D	D	Y	Q	N	N	A
EU498687.1	Italy	2003		FPV-G3C-2	K	M	A	K	V	V	S	A	D	D	Y	Q	N	N	A
EU498716.1	United Kingdom	2007		FPV-G3C-2	K	M	A	K	V	I	S	A	D	D	Y	Q	N	N	A
KX434462.1	Italy	2015		FPV-G3C-2	K	M	A	K	V	I	S	A	D	D	Y	Q	N	N	A
KX943310.1	Italy	2015		FPV-G3C-2	K	M	A	K	V	I	S	A	D	D	Y	Q	N	N	A
KX943314.1	Italy	2015		FPV-G3C-2	K	M	A	K	V	I	S	A	D	D	Y	Q	N	N	A
KX943315.1	Italy	2015		FPV-G3C-2	K	M	A	K	V	I	S	A	D	D	Y	Q	N	N	A
KX943316.1	Italy	2015		FPV-G3C-2	K	M	A	K	V	I	S	A	D	D	Y	Q	N	N	A
KX943317.1	Italy	2015		FPV-G3C-2	K	M	A	K	V	I	S	A	D	D	Y	Q	N	N	A
M10824.1	USA	1985		FPV-G3C-2	K	M	A	K	V	I	S	A	D	D	Y	Q	N	N	A
MK570644.1	Australia	2015		FPV-G3C-2	K	M	A	K	V	I	S	A	D	D	Y	Q	N	N	A
MK570645.1	Australia	2015		FPV-G3C-2	K	M	A	K	V	I	S	A	D	D	Y	Q	N	N	A
MN127780.1	Thailand	2019		FPV-G3C-2	K	M	A	K	V	I	S	A	D	D	Y	Q	N	N	A
MN127781.1	Thailand	2019		FPV-G3C-2	K	M	A	K	V	I	S	A	D	D	Y	Q	N	N	A
MW589471.1	Thailand	2020		FPV-G3C-2	K	M	A	K	V	I	S	A	D	D	Y	Q	N	N	A
MW847167.1	Italy	2016		FPV-G3C-2	K	M	A	K	V	I	S	A	D	D	Y	Q	N	N	A
EU498686.1	Italy	2003		FPV-G3C-3	K	M	A	K	V	V	S	A	D	D	Y	Q	N	N	A
EU498692.1	Italy	2004		FPV-G3C-3	K	M	A	K	V	V	S	A	D	D	Y	Q	N	N	A
KT240128.1	Portugal	2006		FPV-G3C-3	K	M	A	K	V	V	S	A	D	D	Y	Q	N	N	A
KT240132.1	Portugal	2012		FPV-G3C-3	K	M	A	K	V	V	S	A	D	D	Y	Q	N	N	A
KU248456.1	Portugal	2006		FPV-G3C-3	K	M	A	K	V	V	S	A	D	D	Y	Q	N	N	A
KU248457.1	Portugal	2006		FPV-G3C-3	K	M	A	K	V	V	S	A	D	D	Y	Q	N	N	A
KU248458.1	Portugal	2006		FPV-G3C-3	K	M	A	K	V	V	S	A	D	D	Y	Q	N	N	A
KU248459.1	Portugal	2006		FPV-G3C-3	K	M	A	K	V	V	S	A	D	D	Y	Q	N	N	A
KU248460.1	Portugal	2006		FPV-G3C-3	K	M	A	K	V	V	S	A	D	D	Y	Q	N	N	A
KU248461.1	Portugal	2006		FPV-G3C-3	K	M	A	K	V	V	S	A	D	D	Y	Q	N	N	A
MK570675.1	Australia	2018		FPV-G3C-3	K	M	A	K	V	V	S	A	D	D	Y	Q	N	N	A
MK570716.1	United Arab Emirate	2017		FPV-G3C-3	K	M	A	K	V	V	S	A	D	D	Y	Q	N	N	A
MK570717.1	United Arab Emirate	2017		FPV-G3C-3	K	M	A	K	V	V	S	A	D	D	Y	Q	N	N	A
MK570718.1	United Arab Emirate	2017		FPV-G3C-3	K	M	A	K	V	V	S	A	D	D	Y	Q	N	N	A
MK570719.1	United Arab Emirate	2017		FPV-G3C-3	K	M	A	K	V	V	S	A	D	D	Y	Q	N	N	A
MK570720.1	United Arab Emirate	2017		FPV-G3C-3	K	M	A	K	V	V	S	A	D	D	Y	Q	N	N	A
MK570721.1	United Arab Emirate	2017		FPV-G3C-3	K	M	A	K	V	V	S	A	D	D	Y	Q	N	N	A
MK570722.1	United Arab Emirate	2017		FPV-G3C-3	K	M	A	K	V	V	S	A	D	D	Y	Q	N	N	A
MK570723.1	United Arab Emirate	2017		FPV-G3C-3	K	M	A	K	V	V	S	A	D	D	Y	Q	N	N	A
MK570724.1	United Arab Emirate	2017		FPV-G3C-3	K	M	A	K	V	V	S	A	D	D	Y	Q	N	N	A
MN603975.1	United Arab Emirate	2017		FPV-G3C-3	K	M	A	K	V	V	S	A	D	D	Y	Q	N	N	A
MN127779.1	Thailand	2018		FPV-G3C-3	K	M	A	K	V	V	S	A	D	D	Y	Q	N	N	A
MW589472.1	Thailand	2020		FPV-G3C-3	K	M	A	K	V	V	S	A	D	D	Y	Q	N	N	A
MW926315.1	United Kingdom	2019		FPV-G3C-3	K	M	A	K	V	V	S	A	D	D	Y	Q	N	N	
OM638043.1	Egypt	2021	canine	FPV-G3C-3	K	M	A	K	V	V	S	A	D	D	Y	Q	N	N	A
OM937916.1	Egypt	2019		FPV-G3C-3	K	M	A	K	V	V	S	A	D	D	Y	Q	N	N	A
EU498705.1	Italy	2006		FPV-G3C-4	K	M	A	K	V	V	S	A	D	D	Y	Q	N	N	A
KX434461.1	Italy	2015		FPV-G3C-4	K	M	A	K	V	V	S	A	D	D	Y	Q	N	N	A
KX943311.1	Italy	2015		FPV-G3C-4	K	M	A	K	V	V	S	A	D	D	Y	Q	N	N	A
KX943312.1	Italy	2015		FPV-G3C-4	K	M	A	K	V	V	S	A	D	D	Y	Q	N	N	A
KX943313.1	Italy	2015		FPV-G3C-4	K	M	A	K	V	V	S	A	D	D	Y	Q	N	N	A
MW847155.1	Italy	2011		FPV-G3C-4	K	M	A	K	V	V	S	A	D	D	Y	Q	N	N	A
MW847158.1	Italy	2011		FPV-G3C-4	K	M	A	K	V	V	S	A	D	D	Y	Q	N	N	A
MW847169.1	Italy	2016		FPV-G3C-4	K	M	A	K	V	V	S	A	D	D	Y	Q	N	N	A
MW847173.1	Italy	2016		FPV-G3C-4	K	M	A	K	V	V	S	A	D	D	Y	Q	N	N	A
MW847174.1	Italy	2016		FPV-G3C-4	K	M	A	K	V	V	S	A	D	D	Y	Q	N	N	

MW847175.1	Italy	2016		FPV-G3C-4	K	M	A	K	V	V	S	A	D	D	Y	Q	N	N	A
MW847176.1	Italy	2016		FPV-G3C-4	K	M	A	K	V	V	S	A	D	D	Y	Q	N	N	A
MW847177.1	Italy	2016		FPV-G3C-4	K	M	A	K	V	V	S	A	D	D	Y	Q	N	N	A
MW847178.1	Italy	2017		FPV-G3C-4	K	M	A	K	V	V	S	A	D	D	Y	Q	N	N	A
MW847179.1	Italy	2017		FPV-G3C-4	K	M	A	K	V	V	S	A	D	D	Y	Q	N	N	A
MW847180.1	Italy	2017		FPV-G3C-4	K	M	A	K	V	V	S	A	D	D	Y	Q	N	N	A
MW847181.1	Italy	2017		FPV-G3C-4	K	M	A	K	V	V	S	A	D	D	Y	Q	N	N	A
MW847183.1	Italy	2017		FPV-G3C-4	K	M	A	K	V	V	S	A	D	D	Y	Q	N	N	A
MW847187.1	Italy	2017		FPV-G3C-4	K	M	A	K	V	V	S	A	D	D	Y	Q	N	N	A
MW847188.1	Italy	2017		FPV-G3C-4	K	M	A	K	V	V	S	A	D	D	Y	Q	N	N	A
MW847189.1	Italy	2017		FPV-G3C-4	K	M	A	K	V	V	S	A	D	D	Y	Q	N	N	A
MW847190.1	Italy	2017		FPV-G3C-4	K	M	A	K	V	V	S	A	D	D	Y	Q	N	N	A
MW847191.1	Italy	2017		FPV-G3C-4	K	M	A	K	V	V	S	A	D	D	Y	Q	N	N	A
MW847192.1	Italy	2017		FPV-G3C-4	K	M	A	K	V	V	S	A	D	D	Y	Q	N	N	A
MW847193.1	Italy	2017		FPV-G3C-4	K	M	A	K	V	V	S	A	D	D	Y	Q	N	N	A
MW847194.1	Italy	2017		FPV-G3C-4	K	M	A	K	V	V	S	A	D	D	Y	Q	N	N	A
MW847199.1	Italy	2018		FPV-G3C-4	K	M	A	K	V	V	S	A	D	D	Y	Q	N	N	A
MW926316.1	United Kingdom	2019		FPV-G3C-4	K	M	A	K	V	V	S	A	D	D	Y	Q	N	N	A
EU498690.1	Italy	2004		FPV-G3C-5	K	M	A	K	V	V	S	A	D	D	Y	Q	N	N	A
EU659115.1	USA	2006		FPV-G3C-5	K	M	A	K	V	V	S	A	D	D	Y	Q	N	N	A
EU498682.1	Italy	2001		FPV-G3	K	M	A	K	V	V	S	A	D	D	Y	Q	N	N	A
EU498704.1	Italy	2006		FPV-G3	K	M	A	K	V	V	S	A	D	D	Y	Q	N	N	A
OP153926.1	South Korea	2019		FPV-G3	K	M	A	K	V	V	S	A	D	D	Y	Q	N	N	A
OP153927.1	South Korea	2019		FPV-G3E	K	M	A	K	V	V	S	A	D	D	Y	Q	N	N	A
OP153930.1	South Korea	2019		FPV-G3E	K	M	A	K	V	V	S	A	D	D	Y	Q	N	N	A
OP153931.1	South Korea	2019		FPV-G3E	K	M	A	K	V	V	S	A	D	D	Y	Q	N	N	A
MZ391096.1	Turkey	2020		FPV-G3F	K	M	A	K	V	V	S	A	D	D	Y	Q	N	N	A
HQ184200.1	South Korea	2008		FPV-G3G	K	M	A	K	V	V	S	A	D	D	Y	Q	N	N	A
MW035309.1	South Korea	2019	canine	FPV-G3G	K	M	A	K	V	V	S	A	D	D	Y	Q	N	N	A
OP153925.1	South Korea	2019		FPV-G3G	K	M	A	K	V	V	S	A	D	D	Y	Q	N	N	A
OP153928.1	South Korea	2019		FPV-G3G	K	M	A	K	V	V	S	A	D	D	Y	Q	N	N	A
OP153932.1	South Korea	2019		FPV-G3G	K	M	A	K	V	V	S	A	D	D	Y	Q	N	N	A
MH559110.1	India	2018		FPV-G3H	K	M	A	K	V	V	S	A	D	D	Y	Q	N	N	A
MN400978.1	South Korea	2017		FPV-G3H	K	M	A	K	V	V	S	A	D	D	Y	Q	N	N	A
MN400980.1	South Korea	2017		FPV-G3H	K	M	A	K	V	V	S	A	D	D	Y	Q	N	N	A
MN683826.1	South Korea	2017		FPV-G3H	K	M	A	K	V	V	S	A	D	D	Y	Q	N	N	A
DQ099431.1	China	2005		FPV-G1	K	M	S	K	V	V	S	A	D	D	Y	Q	N	N	A
MK266797.1	China	2018		FPV-G1	K	M	S	K	V	V	S	A	D	D	Y	Q	N	N	A
MK301396.1	China	2018		FPV-G1	K	M	S	K	V	V	S	A	D	D	Y	Q	N	N	A
MK671169.1	China	2018		FPV-G1	K	M	S	K	V	V	S	A	D	D	Y	Q	N	N	A
MK671179.1	China	2017		FPV-G1	K	M	S	K	V	V	S	A	D	D	Y	Q	N	N	A
MK671184.1	China	2018		FPV-G1	K	M	S	K	V	V	S	A	D	D	Y	Q	N	N	A
MK671185.1	China	2018		FPV-G1	K	M	S	K	V	V	S	A	D	D	Y	Q	N	N	A
MN418999.1	China	2018		FPV-G1	K	M	S	K	V	V	S	A	D	D	Y	Q	N	N	A
MN419012.1	China	2019		FPV-G1	K	M	S	K	V	V	S	A	D	D	Y	Q	N	N	A
MT270531.1	China	2019		FPV-G1	K	M	S	K	V	V	S	A	D	D	Y	Q	N	N	A
MT270532.1	China	2019		FPV-G1	K	M	S	K	V	V	S	A	D	D	Y	Q	N	N	A
MT270533.1	China	2019		FPV-G1	K	M	S	K	V	V	S	A	D	D	Y	Q	N	N	A
MT270534.1	China	2019		FPV-G1	K	M	A	K	V	V	S	A	D	D	Y	Q	N	N	A
MT270535.1	China	2019		FPV-G1	K	M	S	K	V	V	S	A	D	D	Y	Q	N	N	A
MT270536.1	China	2019		FPV-G1	K	M	S	K	V	V	S	A	D	D	Y	Q	N	N	A
MT270537.1	China	2019		FPV-G1	K	M	S	K	V	V	S	A	D	D	Y	Q	N	N	A
MT270538.1	China	2019		FPV-G1	K	M	S	K	V	V	S	A	D	D	Y	Q	N	N	A
MT270539.1	China	2019		FPV-G1	K	M	S	K	V	V	S	A	D	D	Y	Q	N	N	A
MT270540.1	China	2019		FPV-G1	K	M	S	K	V	V	S	A	D	D	Y	Q	N	N	A
MT270541.1	China	2019		FPV-G1	K	M	S	K	V	V	S	A	D	D	Y	Q	N	N	A
MT270542.1	China	2019		FPV-G1	K	M	S	K	V	V	S	A	D	D	Y	Q	N	N	A
MT270544.1	China	2019		FPV-G1	K	M	S	K	V	V	S	A	D	D	Y	Q	N	N	A
MT270547.1	China	2019		FPV-G1	K	M	S	K	V	V	S	A	D	D	Y	Q	N	N	A
MT270548.1	China	2019		FPV-G1	K	M	S	K	V	V	S	A	D	D	Y	Q	N	N	A
MT270550.1	China	2019		FPV-G1	K	M	S	K	V	V	S	A	D	D	Y	Q	N	N	A
MT270551.1	China	2019		FPV-G1	K	M	S	K	V	V	S	A	D	D	Y	Q	N	N	A
MT270552.1	China	2019		FPV-G1	K	M	S	K	V	V	S	A	D	D	Y	Q	N	N	A
MT270553.1	China	2019		FPV-G1	K	M	S	K	V	V	S	A	D	D	Y	Q	N	N	A
MT270554.1	China	2019		FPV-G1	K	M	S	K	V	V	S	A	D	D	Y	Q	N	N	A
MT270555.1	China	2019		FPV-G1	K	M	S	K	V	V	S	A	D	D	Y	Q	N	N	A
MT270556.1	China	2019		FPV-G1	K	M	S	K	V	V	S	A	D	D	Y	Q	N	N	A
MT270557.1	China	2019		FPV-G1	K	M	S	K	V	V	S	A	D	D	Y	Q	N	N	A
MT270558.1	China	2019		FPV-G1	K	M	S	K	V	V	S	A	D	D	Y	Q	N	N	A
MT270559.1	China	2019		FPV-G1	K	M	S	K	V	V	S	A	D	D	Y	Q	N	N	A
MT270560.1	China	2019		FPV-G1	K	M	S	K	V	V	S	A	D	D	Y	Q	N	N	A
MT270561.1	China	2019		FPV-G1	K	M	S	K	V	V	S	A	D	D	Y	Q	N	N	A
MT270562.1	China	2019		FPV-G1	K	M	S	K	V	V	S	A	D	D	Y	Q	N	N	A
MT270565.1	China	2019		FPV-G1	K	M	S	K	V	V	S	A	D	D	Y	Q	N	N	A
MT270568.1	China	2019		FPV-G1	K	M	S	K	V	V	S	A	D	D	Y	Q	N	N	A
MT270569.1	China	2019		FPV-G1	K	M	S	K	V	V	S	A	D	D	Y	Q	N	N	A
MT270571.1	China	2019		FPV-G1	K	M	S	K	V	V	S	G	D	D	Y	Q	E	S	G
MT270572.1	China	2019		FPV-G1	K	M	S	K	V	V	S	A	D	D	Y	Q	N	N	A
MT270573.1	China	2019		FPV-G1	K	M	S	K	V	V	S	A	D	D	Y	Q	N	N	A
MT270574.1	China	2019		FPV-G1	K	M	S	K	V	V	S	A	D	D	Y	Q	N	N	A
MT270575.1	China	2019		FPV-G1	K	M	S	K	V	V	S	A	D	D	Y	Q	N	N	A
MT270576.1	China	2019		FPV-G1	K	M	S	K	V	V	S	A	D	D	Y	Q	N	N	A
MT270579.1	China	2019		FPV-G1	K	M	S	K	V	V	S	A	D	D	Y	Q	N	N	A
MT270580.1	China	2019		FPV-G1	K	M	S	K	V	V	S	A	D	D	Y	Q	N	N	A
MT270581.1	China	2019		FPV-G1	K	M	S	K	V	V	S	A	D	D	Y	Q	N	N	A
MT614366.1	China	2019		FPV-G1	K	M	S	K	V	V	S	A	D	D	Y	Q	N	N	A
MT892650.1	China	2019		FPV-G1	K	M	S	K	V	V	S	A	D	D	Y	Q	N	N	A
MW017596.1	China	2019	canine	FPV-G1	K	M	S	K	V	V	S	A	D	D	Y	Q	N	N	A
MW017616.1	China	2020	canine	FPV-G1	K	M	S	K	V	V	S	A	D	D	Y	Q	N	N	A
MW017618.1	China	2020	canine	FPV-G1	K	M	S	K	V	V	S	A	D	D	Y	Q	N	N	A
MW017626.1	China	2020		FPV-G1	K	M	S	K	V	V	S	A	D	D	Y	Q	N	N	A
MW017629.1	China	2020		FPV-G1	K	M	S	K	V	V	S	A	D	D	Y	Q	N	N	A

MW017630.1	China	2020		FPV-G1	K	M	S	K	V	V	S	A	D	D	Y	Q	N	N	A
MW017631.1	China	2020		FPV-G1	K	M	S	K	V	V	S	A	D	D	Y	Q	N	N	A
MW495829.1	China	2018		FPV-G1	K	M	S	K	V	V	S	A	D	D	Y	Q	N	N	A
MW495832.1	China	2019		FPV-G1	K	M	S	K	V	V	S	A	D	D	Y	Q	N	N	A
MW495836.1	China	2019		FPV-G1	K	M	S	K	V	V	S	A	D	D	Y	Q	N	N	A
MW495837.1	China	2019		FPV-G1	K	M	S	K	V	V	S	A	D	D	Y	Q	N	N	A
MW495839.1	China	2018		FPV-G1	K	M	S	K	V	V	S	A	D	D	Y	Q	N	N	A
MW495840.1	China	2018		FPV-G1	K	M	S	K	V	V	S	A	D	D	Y	Q	N	N	A
MW495841.1	China	2019		FPV-G1	K	M	S	K	V	V	S	A	D	D	Y	Q	N	N	A
MW495842.1	China	2020		FPV-G1	K	M	S	K	V	V	S	A	D	D	Y	Q	N	N	A
MW495843.1	China	2020		FPV-G1	K	M	S	K	V	V	S	A	D	D	Y	Q	N	N	A
MW495844.1	China	2021		FPV-G1	K	M	S	K	V	V	S	A	D	D	Y	Q	N	N	A
MW495845.1	China	2020		FPV-G1	K	M	S	K	V	V	S	A	D	D	Y	Q	N	N	A
MW495846.1	China	2020		FPV-G1	K	M	S	K	V	V	S	A	D	D	Y	Q	N	N	A
MW495847.1	China	2020		FPV-G1	K	M	S	K	V	V	S	A	D	D	Y	Q	N	N	A
MW650831.1	China	2020		FPV-G1	K	M	S	K	V	V	S	A	D	D	Y	Q	N	N	A
MW659466.1	China	2020		FPV-G1	K	M	S	K	V	V	S	A	D	D	Y	Q	N	N	A
MW791426.1	China	2020		FPV-G1	K	M	S	K	V	V	S	A	D	D	Y	Q	N	N	A
MW791427.1	China	2020		FPV-G1	K	M	S	K	V	V	S	A	D	D	Y	Q	N	N	A
MW811187.1	China	2020		FPV-G1	K	M	S	K	V	V	S	A	D	D	Y	Q	N	N	A
MZ357120.1	China	2020		FPV-G1	K	M	S	K	V	V	S	A	D	D	Y	Q	N	N	A
MZ442302.1	China	2020		FPV-G1	K	M	S	K	V	V	S	A	D	D	Y	Q	N	N	A
MZ442312.1	China	2017		FPV-G1	K	M	S	K	V	V	S	A	D	D	Y	Q	N	N	A
MZ442313.1	China	2020		FPV-G1	K	M	S	K	V	V	S	A	D	D	Y	Q	N	N	A
MZ442314.1	China	2020		FPV-G1	K	M	S	K	V	V	S	A	D	D	Y	Q	N	N	A
MZ836347.1	China	2019		FPV-G1	K	M	S	K	V	V	S	A	D	D	Y	Q	N	N	A
MZ836350.1	China	2020		FPV-G1	K	M	S	K	V	V	S	A	D	D	Y	Q	N	N	A
MZ836352.1	China	2020		FPV-G1	K	M	S	K	V	V	S	A	D	D	Y	Q	N	N	A
MZ836353.1	China	2020		FPV-G1	K	M	S	K	V	V	S	A	D	D	Y	Q	N	N	A
MZ836354.1	China	2020		FPV-G1	K	M	S	K	V	V	S	A	D	D	Y	Q	N	N	A
MZ836355.1	China	2020		FPV-G1	K	M	S	K	V	V	S	A	D	D	Y	Q	N	N	A
MZ836356.1	China	2020		FPV-G1	K	M	S	K	V	V	S	A	D	D	Y	Q	N	N	A
MZ836358.1	China	2020		FPV-G1	K	M	S	K	V	V	S	A	D	D	Y	Q	N	N	A
MZ836361.1	China	2020		FPV-G1	K	M	S	K	V	V	S	A	D	D	Y	Q	N	N	A
MZ836363.1	China	2020		FPV-G1	K	M	S	K	V	V	S	A	D	D	Y	Q	N	N	A
MZ836364.1	China	2020		FPV-G1	K	M	S	K	V	V	S	A	D	D	Y	Q	N	N	A
MZ836367.1	China	2020		FPV-G1	K	M	S	K	V	V	S	A	D	D	Y	Q	N	N	A
MZ836368.1	China	2020		FPV-G1	K	M	S	K	V	V	S	A	D	D	Y	Q	N	N	A
MZ836373.1	China	2020		FPV-G1	K	M	S	K	V	V	S	A	D	D	Y	Q	N	N	A
OK128324.1	China	2019	canine	FPV-G1	K	M	S	K	V	V	S	A	D	D	Y	Q	N	N	A
OK128325.1	China	2021	canine	FPV-G1	K	M	S	K	V	V	S	G	D	D	Y	Q	N	N	A
OK384310.1	China	2019		FPV-G1	K	M	S	K	V	V	S	A	D	D	Y	Q	N	N	G
OK384311.1	China	2019		FPV-G1	K	M	S	K	V	V	S	A	D	D	Y	Q	N	S	G
OK384312.1	China	2019		FPV-G1	K	M	S	K	V	V	S	A	D	D	Y	Q	N	N	G
OK384313.1	China	2019		FPV-G1	K	M	S	K	V	V	S	A	D	D	Y	Q	N	S	G
OK384314.1	China	2019		FPV-G1	K	M	S	K	V	V	S	A	D	D	Y	Q	N	S	G
OL547731.1	China	2021		FPV-G1	K	M	S	K	V	V	S	A	D	D	Y	Q	N	N	A
OL547732.1	China	2020		FPV-G1	K	M	S	K	V	V	S	A	D	D	Y	Q	N	N	A
OL547733.1	China	2019		FPV-G1	K	M	S	K	V	V	S	A	D	D	Y	Q	N	N	A
OL547734.1	China	2020		FPV-G1	K	M	S	K	V	V	S	A	D	D	Y	Q	N	N	A
OL547735.1	China	2019		FPV-G1	K	M	S	K	V	V	S	A	D	D	Y	Q	N	N	A
OL547736.1	China	2019		FPV-G1	K	M	S	K	V	V	S	A	D	D	Y	Q	N	N	A
OL547737.1	China	2020		FPV-G1	K	M	S	K	V	V	S	A	D	D	Y	Q	N	N	A
OM212011.1	China	2021		FPV-G1	K	M	S	K	V	V	S	A	D	D	Y	Q	N	N	A
OM885373.1	China	2021		FPV-G1	K	M	S	K	V	V	S	A	D	D	Y	Q	N	N	A
OM885374.1	China	2021		FPV-G1	K	M	S	K	V	V	S	A	D	D	Y	Q	N	N	A
OM885376.1	China	2021		FPV-G1	K	M	S	K	V	V	S	A	D	D	Y	Q	N	N	A
OM885377.1	China	2021		FPV-G1	K	M	L	K	V	V	S	A	D	D	Y	Q	N	N	A
OM885378.1	China	2021		FPV-G1	K	M	S	K	V	V	S	A	D	D	Y	Q	N	N	A
OM885379.1	China	2021		FPV-G1	K	M	S	K	V	V	S	A	D	D	Y	Q	N	N	A
OM885380.1	China	2021		FPV-G1	K	M	S	K	V	V	S	A	D	D	Y	Q	N	N	A
OM885382.1	China	2021		FPV-G1	K	M	S	K	V	V	S	A	D	D	Y	Q	N	N	A
OM885383.1	China	2021		FPV-G1	K	M	S	K	V	V	S	A	D	D	Y	Q	N	N	A
OM885384.1	China	2021		FPV-G1	K	M	S	K	V	V	S	A	D	D	Y	Q	N	N	A
OM918771.1	China	2021		FPV-G1	K	M	S	K	V	V	S	A	D	D	Y	Q	N	N	A
OM918772.1	China	2021		FPV-G1	K	M	S	K	V	V	S	A	D	D	Y	Q	N	N	A
OM918774.1	China	2021		FPV-G1	K	M	S	K	V	V	S	A	D	D	Y	Q	N	N	A
OM918775.1	China	2021		FPV-G1	K	M	S	K	V	V	S	A	D	D	Y	Q	N	N	A
OM918776.1	China	2021		FPV-G1	K	M	L	K	V	V	S	A	D	D	Y	Q	N	N	A
OM918777.1	China	2021		FPV-G1	K	M	S	K	V	V	S	A	D	D	Y	Q	N	N	A
OM918778.1	China	2021		FPV-G1	K	M	S	K	V	V	S	A	D	D	Y	Q	N	N	A
OM918779.1	China	2021		FPV-G1	K	M	S	K	V	V	S	A	D	D	Y	Q	N	N	A
OM918780.1	China	2021		FPV-G1	K	M	S	K	V	V	S	A	D	D	Y	Q	N	N	A
OM918782.1	China	2021		FPV-G1	K	M	S	K	V	V	S	A	D	D	Y	Q	N	N	A
OM918783.1	China	2021		FPV-G1	K	M	S	K	V	V	S	A	D	D	Y	Q	N	N	A
OM918784.1	China	2021		FPV-G1	K	M	S	K	V	V	S	A	D	D	Y	Q	N	N	A
OM918785.1	China	2021		FPV-G1	K	M	S	K	V	V	S	A	D	D	Y	Q	N	N	A
OP313105.1	China	2020		FPV-G1	K	M	S	K	V	V	S	A	D	D	Y	Q	N	N	A
OP471917.1	China	2019		FPV-G1	K	M	S	K	V	V	S	A	D	D	Y	Q	N	N	A
OP471918.1	China	2019		FPV-G1	K	M	S	K	V	V	S	A	D	D	Y	Q	N	N	A
ON646205.1	China	2018		FPV-G1	K	M	S	K	V	V	S	A	D	D	Y	Q	N	N	A
ON646206.1	China	2018		FPV-G1	K	M	A	K	V	V	S	A	D	D	Y	Q	N	N	A
ON646207.1	China	2018		FPV-G1	K	M	A	K	V	V	S	A	D	D	Y	Q	N	N	A
ON646209.1	China	2019		FPV-G1	K	M	S	K	V	V	S	A	D	D	Y	Q	N	N	A
ON646211.1	China	2019		FPV-G1	K	M	A	K	V	I	S	A	D	D	Y	Q	N	N	A
ON646215.1	China	2019		FPV-G1	K	M	A	K	V	V	S	A	D	D	Y	Q	N	N	A
AF015223.1	Taiwan China	1998		FPV-G2	K	M	A	K	V	I	S	A	D	D	Y	Q	N	N	A
DQ099430.1	China	2005		FPV-G3A	K	M	A	K	V	V	S	A	D	D	Y	Q	N	N	A
DQ474235.1	China	2006		FPV-G3A	K	M	A	K	V	V	S	A	D	D	Y	Q	N	N	A
DQ474236.1	China	2006		FPV-G3A	K	M	A	K	V	V	S	A	D	D	Y	Q	N	N	A
DQ474237.1	China	2006		FPV-G3A	K	M	A	K	V	V	S	A	D	D	Y	Q	N	N	A
DQ474238.1	China	2006		FPV-G3A	K	M	A	K	V	I	S	A	D	D	Y	Q	N	N	A

EF988660.1	China	2007	FPV-G3A	K	M	A	K	V	V	S	A	D	D	Y	Q	N	N	A
FJ936171.1	China	2008	FPV-G3A	K	M	A	K	V	I	S	A	D	D	Y	Q	N	N	A
KP280068.1	China	2014	FPV-G3A	K	M	A	K	V	V	S	A	D	D	Y	Q	N	N	A
MF541119.1	China	2016/2017	FPV-G3A	K	M	A	K	V	V	S	A	D	D	Y	Q	N	N	A
MF541124.1	China	2016/2017	FPV-G3A	K	M	A	K	V	V	S	A	D	D	Y	Q	N	N	A
MF541126.1	China	2016/2017	FPV-G3A	K	M	A	K	V	V	S	A	D	D	Y	Q	N	S	G
MF541128.1	China	2016/2017	FPV-G3A	K	M	A	K	V	V	S	A	D	D	Y	Q	N	N	A
MF541130.1	China	2016/2017	FPV-G3A	K	M	A	K	V	V	S	A	D	D	Y	Q	N	N	A
MF541131.1	China	2016/2017	FPV-G3A	K	M	A	K	V	V	S	A	D	D	Y	Q	N	N	A
MF541132.1	China	2016/2017	FPV-G3A	K	M	A	K	V	V	S	A	D	D	Y	Q	N	N	A
MF541133.1	China	2016/2017	FPV-G3A	K	M	A	K	V	V	S	A	D	D	Y	Q	N	N	A
MF541134.1	China	2016/2017	FPV-G3A	K	M	A	K	V	V	S	A	D	D	Y	Q	N	N	A
MF541136.1	China	2016/2017	FPV-G3A	K	M	A	K	V	V	S	A	D	D	Y	Q	N	N	A
MF541137.1	China	2016/2017	FPV-G3A	K	M	A	K	V	V	S	A	D	D	Y	Q	N	N	A
MF541138.1	China	2016/2017	FPV-G3A	K	M	A	K	V	V	S	A	D	D	Y	Q	N	N	A
MF541140.1	China	2016/2017	FPV-G3A	K	M	A	K	V	V	S	A	D	D	Y	Q	N	N	A
MG924893.1	China	2018	FPV-G3A	K	M	A	K	V	V	S	A	D	D	Y	Q	N	N	A
MH329286.1	China	2016	FPV-G3A	K	M	A	K	V	V	S	A	D	D	Y	Q	N	N	A
MK266786.1	China	2017	FPV-G3A	K	M	A	K	V	V	S	A	D	D	Y	Q	N	N	A
MK266787.1	China	2018	FPV-G3A	K	M	A	K	V	V	S	A	D	D	Y	Q	N	N	A
MK266788.1	China	2017	FPV-G3A	K	M	A	K	V	V	S	A	D	D	Y	Q	N	N	A
MK266789.1	China	2018	FPV-G3A	K	M	A	K	V	V	S	A	D	D	Y	Q	N	N	A
MK266790.1	China	2017	FPV-G3A	K	M	A	K	V	V	S	A	D	D	Y	Q	N	N	A
MK266791.1	China	2017	FPV-G3A	K	M	A	K	V	V	S	A	D	D	Y	Q	N	N	A
MK266792.1	China	2017	FPV-G3A	K	M	A	K	V	V	S	A	D	D	Y	Q	N	N	A
MK266795.1	China	2017	FPV-G3A	K	M	A	K	V	V	S	A	D	D	Y	Q	N	N	A
MK266796.1	China	2018	FPV-G3A	K	M	A	K	V	V	S	A	D	D	Y	Q	N	N	A
MK295775.1	China	2017	FPV-G3A	K	M	A	K	V	V	S	A	D	D	Y	Q	N	N	A
MK671151.1	China	2016	FPV-G3A	K	M	A	K	V	V	S	A	D	D	Y	Q	N	N	A
MK671153.1	China	2016	FPV-G3A	K	M	A	K	V	V	S	A	D	D	Y	Q	N	N	A
MK671155.1	China	2016	FPV-G3A	K	M	A	K	V	V	S	A	D	D	Y	Q	N	N	A
MK671156.1	China	2016	FPV-G3A	K	M	A	K	V	V	S	A	D	D	Y	Q	N	N	A
MK671157.1	China	2017	FPV-G3A	K	M	A	K	V	V	S	A	D	D	Y	Q	N	N	A
MK671158.1	China	2017	FPV-G3A	K	M	A	K	V	V	S	A	D	D	Y	Q	N	N	A
MK671160.1	China	2017	FPV-G3A	K	M	A	K	V	V	S	A	D	D	Y	Q	N	N	A
MK671162.1	China	2017	FPV-G3A	K	M	A	K	V	V	S	A	D	D	Y	Q	N	N	A
MK671163.1	China	2017	FPV-G3A	K	M	A	K	V	V	S	A	D	D	Y	Q	N	N	A
MK671164.1	China	2017	FPV-G3A	K	M	S	K	V	V	S	A	D	D	Y	Q	N	N	A
MK671170.1	China	2017	FPV-G3A	K	M	A	K	V	V	S	A	D	D	Y	Q	N	N	A
MK671171.1	China	2017	FPV-G3A	K	M	A	K	V	V	S	A	D	D	Y	Q	N	N	A
MK671172.1	China	2018	FPV-G3A	K	M	A	K	V	V	S	A	D	D	Y	Q	N	N	A
MK671173.1	China	2018	FPV-G3A	K	M	A	K	V	V	S	A	D	D	Y	Q	N	N	A
MK671174.1	China	2018	FPV-G3A	K	M	A	K	V	V	S	A	D	D	Y	Q	N	N	A
MK671175.1	China	2018	FPV-G3A	K	M	S	K	V	V	S	A	D	D	Y	Q	N	N	A
MK671176.1	China	2018	FPV-G3A	K	M	A	K	V	V	S	A	D	D	Y	Q	N	N	A
MK671177.1	China	2018	FPV-G3A	K	M	A	K	V	V	S	A	D	D	Y	Q	N	N	A
MK671180.1	China	2018	FPV-G3A	K	M	A	K	V	V	S	A	D	D	Y	Q	N	N	A
MK671181.1	China	2018	FPV-G3A	K	M	A	K	V	V	S	A	D	D	Y	Q	N	N	A
MK671182.1	China	2018	FPV-G3A	K	M	A	K	V	V	S	A	D	D	Y	Q	N	N	A
MK671183.1	China	2018	FPV-G3A	K	M	A	K	V	V	S	A	D	D	Y	Q	N	N	A
MK671186.1	China	2018	FPV-G3A	K	M	A	K	V	V	S	A	D	D	Y	Q	N	N	A
MK671187.1	China	2018	FPV-G3A	K	M	A	K	V	V	S	A	D	D	Y	Q	N	N	A
MK671188.1	China	2018	FPV-G3A	K	M	A	K	V	V	S	A	D	D	Y	Q	N	N	A
MN418997.1	China	2018	FPV-G3A	K	M	A	K	V	V	S	A	D	D	Y	Q	N	N	A
MN418998.1	China	2018	FPV-G3A	K	M	A	K	V	V	S	A	D	D	Y	Q	N	N	A
MN419003.1	China	2019	FPV-G3A	K	M	A	K	V	V	S	A	D	D	Y	Q	N	N	A
MN419004.1	China	2019	FPV-G3A	K	M	A	K	V	V	S	A	D	D	Y	Q	N	N	A
MN419006.1	China	2019	FPV-G3A	K	M	A	K	V	V	S	A	D	D	Y	Q	N	N	A
MN419007.1	China	2019	FPV-G3A	K	M	A	K	V	V	S	A	D	D	Y	Q	N	N	A
MN419008.1	China	2019	FPV-G3A	K	M	A	K	V	V	S	A	D	D	Y	Q	N	N	A
MN419009.1	China	2019	FPV-G3A	K	M	A	K	V	V	S	A	D	D	Y	Q	N	N	A
MN419010.1	China	2019	FPV-G3A	K	M	A	K	V	V	S	A	D	D	Y	Q	N	N	A
MN419011.1	China	2019	FPV-G3A	K	M	A	K	V	V	S	A	D	D	Y	Q	N	N	A
MN419013.1	China	2019	FPV-G3A	K	M	A	K	V	V	S	A	D	D	Y	Q	N	N	A
MT270546.1	China	2019	FPV-G3A	K	M	A	K	V	V	S	A	D	D	Y	Q	N	N	A
MT270549.1	China	2019	FPV-G3A	K	M	A	K	V	V	S	A	D	D	Y	Q	N	N	A
MT270577.1	China	2019	FPV-G3A	K	M	A	K	V	V	S	A	D	D	Y	Q	N	N	A
MT270582.1	China	2019	FPV-G3A	K	M	A	K	V	V	S	A	D	D	Y	Q	N	N	A
MT270583.1	China	2019	FPV-G3A	K	M	A	K	V	V	S	A	D	D	Y	Q	N	N	A
MT270584.1	China	2019	FPV-G3A	K	M	A	K	V	V	S	A	D	D	Y	Q	N	N	A
MW017625.1	China	2020	FPV-G3A	K	M	A	K	V	V	S	A	D	D	Y	Q	N	N	A
MW017627.1	China	2020	FPV-G3A	K	M	A	K	V	V	S	A	D	D	Y	Q	N	N	A
MW495830.1	China	2018	FPV-G3A	K	M	A	K	V	V	S	A	D	D	Y	Q	N	N	A
MW495833.1	China	2017	FPV-G3A	K	M	A	K	V	V	S	A	D	D	Y	Q	N	N	A
MZ442303.1	China	2019	FPV-G3A	K	M	A	K	V	V	S	A	D	D	Y	Q	N	N	A
MZ442304.1	China	2019	FPV-G3A	K	M	A	K	V	V	S	A	D	D	Y	Q	N	N	A
MZ442306.1	China	2018	FPV-G3A	K	M	A	K	V	V	S	A	D	D	Y	Q	N	N	A
MZ442311.1	China	2018	FPV-G3A	K	M	A	K	V	V	S	A	D	D	Y	Q	N	N	A
MZ836351.1	China	2020	FPV-G3A	K	M	A	K	V	V	S	A	D	D	Y	Q	N	N	A
MZ913313.1	China	2021	FPV-G3A	K	M	A	K	V	V	S	A	D	D	Y	Q	N	N	A
OM885375.1	China	2021	FPV-G3A	K	M	A	K	V	V	S	A	D	D	Y	Q	N	N	A
OM885381.1	China	2021	FPV-G3A	K	M	A	K	V	V	S	A	D	D	Y	Q	N	N	A
ON646208.1	China	2019	FPV-G3A	K	M	S	K	V	V	S	A	D	D	Y	Q	N	N	A
ON646201.1	China	2018	FPV-G3A	K	M	A	K	V	V	S	A	D	D	Y	Q	N	N	A
ON646202.1	China	2018	FPV-G3A	K	M	A	K	V	V	S	A	D	D	Y	Q	N	N	A
ON646203.1	China	2018	FPV-G3A	K	M	A	K	V	V	S	A	D	D	Y	Q	N	N	A
ON646210.1	China	2019	FPV-G3A	K	M	A	K	V	V	S	A	D	D	Y	Q	N	N	A
ON646212.1	China	2019	FPV-G3A	K	M	A	K	V	V	S	A	D	D	Y	Q	N	N	A
ON646213.1	China	2019	FPV-G3A	K	M	A	K	V	V	A	A	D	D	Y	Q	N	N	A
ON646214.1	China	2019	FPV-G3A	K	M	A	K	V	V	S	A	D	D	Y	Q	N	N	A
ON646216.1	China	2019	FPV-G3A	K	M	A	K	V	V	S	A	D	D	Y	Q	N	N	A
ON646217.1	China	2019	FPV-G3A	K	M	A	K	V	I	S	A	D	D	Y	Q	N	N	A

ON646218.1	China	2019		FPV-G3A	K	M	A	K	V	I	S	A	D	D	Y	Q	N	N	A
OP471919.1	China	2019		FPV-G3A	K	M	A	K	V	V	S	A	D	D	Y	Q	N	N	A
MH165481.1	China	2015		FPV-G3C-2	K	M	A	K	V	I	S	A	D	D	Y	Q	N	N	A
MH165482.1	China	2014		FPV-G3C-2	K	M	A	K	V	I	S	A	D	D	Y	Q	N	N	A
MK266799.1	China	2018		FPV-G3C-2	K	M	A	K	V	V	S	A	D	D	Y	Q	D	S	G
MN419005.1	China	2019		FPV-G3C-2	K	M	A	K	V	I	S	A	D	D	Y	Q	N	N	A
MT270545.1	China	2019		FPV-G3C-2	K	M	A	K	V	V	S	A	D	D	Y	Q	N	N	A
MT270567.1	China	2019		FPV-G3C-2	K	M	A	K	V	V	S	A	D	D	Y	Q	N	N	A
MT270570.1	China	2019		FPV-G3C-2	K	M	A	K	V	V	S	A	D	D	Y	Q	N	N	A
MT270578.1	China	2019		FPV-G3C-2	K	M	A	K	V	V	S	A	D	D	Y	Q	N	N	A
MW495831.1	China	2017		FPV-G3C-2	K	M	A	K	V	V	S	A	D	D	Y	Q	N	N	A
MW495834.1	China	2018		FPV-G3C-2	K	M	A	K	V	V	S	A	D	D	Y	Q	N	N	A
MW495848.1	China	2020		FPV-G3C-2	K	M	A	K	V	V	S	A	D	D	Y	Q	N	N	A
MF541120.1	China	2016/2017		FPV-G3C4	K	M	A	K	V	V	S	A	D	D	Y	Q	N	N	A
MF541121.1	China	2016/2017		FPV-G3C4	K	M	A	K	V	V	S	A	D	D	Y	Q	N	N	A
MF541123.1	China	2016/2017		FPV-G3C4	K	M	A	K	V	V	S	A	D	D	Y	Q	N	N	A
MF541125.1	China	2016/2017		FPV-G3C4	K	M	A	K	V	V	S	A	D	D	Y	Q	N	N	A
MK266798.1	China	2018		FPV-G3C4	K	M	A	K	V	V	S	A	D	D	Y	Q	N	N	A
MK671154.1	China	2016		FPV-G3C4	K	M	A	K	V	V	S	A	D	D	Y	Q	N	N	A
MK671159.1	China	2017		FPV-G3C4	K	M	S	K	V	V	S	A	D	D	Y	Q	N	N	A
MK671167.1	China	2017		FPV-G3C4	K	M	A	K	V	V	S	A	D	D	Y	Q	N	N	A
MK671178.1	China	2018		FPV-G3C4	K	M	A	K	V	V	S	A	D	D	Y	Q	N	N	A
MN419000.1	China	2018		FPV-G3C4	K	M	A	K	V	V	S	A	D	D	Y	Q	N	N	A
MZ836357.1	China	2020		FPV-G3C4	K	M	A	K	V	V	S	A	D	D	Y	Q	N	N	A
MF541122.1	China	2016/2017		FPV-G3D	K	M	A	K	V	V	S	A	D	D	Y	Q	N	N	A
MF541127.1	China	2016/2017		FPV-G3D	K	M	A	K	V	V	S	A	D	D	Y	Q	N	N	A
MF541129.1	China	2016/2017		FPV-G3D	K	M	A	K	V	V	S	A	D	D	Y	Q	N	N	A
MF541135.1	China	2016/2017		FPV-G3D	K	M	A	K	V	V	S	A	D	D	Y	Q	N	N	A
MF541139.1	China	2016/2017		FPV-G3D	K	M	A	K	V	V	S	A	D	D	Y	Q	N	N	A
MK266782.1	China	2017		FPV-G3D	K	M	A	K	V	V	S	A	D	D	Y	Q	N	N	A
MK266783.1	China	2018		FPV-G3D	K	M	A	K	V	V	S	A	D	D	Y	Q	N	N	A
MK266784.1	China	2017		FPV-G3D	K	M	A	K	V	V	S	A	D	D	Y	Q	N	N	A
MK266785.1	China	2017		FPV-G3D	K	M	A	K	V	V	S	A	D	D	Y	Q	N	N	A
MK671150.1	China	2016		FPV-G3D	K	M	A	K	V	V	S	A	D	D	Y	Q	N	N	A
MK671152.1	China	2016		FPV-G3D	K	M	A	K	V	V	S	A	D	D	Y	Q	N	N	A
MK671161.1	China	2017		FPV-G3D	K	M	A	K	V	V	S	A	D	D	Y	Q	N	N	A
MK671165.1	China	2017		FPV-G3D	K	M	A	K	V	V	S	A	D	D	Y	Q	N	N	A
MK671166.1	China	2017		FPV-G3D	K	M	A	K	V	V	S	A	D	D	Y	Q	N	N	A
MK671168.1	China	2017		FPV-G3D	K	M	A	K	V	V	S	A	D	D	Y	Q	N	N	A
JX048608.1	Taiwan China	2001		FPV-G3F	K	M	A	K	V	V	S	A	D	D	Y	Q	N	N	A
KC473946.1	China	2012		FPV-G3F	K	M	A	K	V	V	S	A	D	D	Y	Q	N	N	A
MK266793.1	China	2018		FPV-G3F	K	M	A	K	V	V	S	A	D	D	Y	Q	N	N	A
MK266794.1	China	2018		FPV-G3F	K	M	A	K	V	V	S	A	D	D	Y	Q	N	N	A
MT270543.1	China	2019		FPV-G3F	K	M	A	K	V	V	S	A	D	D	Y	Q	N	N	A
MW017628.1	China	2020		FPV-G3F	K	M	A	K	V	V	S	A	D	D	Y	Q	N	N	A
MW495835.1	China	2018		FPV-G3F	K	M	A	K	V	V	S	A	D	D	Y	Q	N	N	A
MW495838.1	China	2017		FPV-G3F	K	M	A	K	V	V	S	A	D	D	Y	Q	N	N	A
MZ836362.1	China	2020		FPV-G3F	K	M	A	K	V	V	S	A	D	D	Y	Q	N	N	A
MZ836365.1	China	2020		FPV-G3F	K	M	A	K	V	V	S	A	D	D	Y	Q	N	N	A
MZ836366.1	China	2020		FPV-G3F	K	M	A	K	V	V	S	A	D	D	Y	Q	N	N	A
MZ836376.1	China	2020		FPV-G3F	K	M	A	K	V	V	S	A	D	D	Y	Q	N	N	A
MZ836377.1	China	2020		FPV-G3F	K	M	A	K	V	V	S	A	D	D	Y	Q	N	N	A
MZ836378.1	China	2019		FPV-G3F	K	M	A	K	V	V	S	A	D	D	Y	Q	N	N	A
OM918773.1	China	2021		FPV-G3F	K	M	A	K	V	V	S	A	D	D	Y	Q	N	N	A
MN419001.1	China	2018		FPV-G3G	K	M	A	K	V	V	S	A	D	D	Y	Q	N	N	A
MN419002.1	China	2018		FPV-G3G	K	M	A	K	V	V	S	A	D	D	Y	Q	N	N	A
MT270563.1	China	2019		FPV-G3G	K	M	A	K	V	V	S	A	D	D	Y	Q	N	N	A
MT270564.1	China	2019		FPV-G3G	K	M	A	K	V	V	S	A	D	D	Y	Q	N	N	A
MT270566.1	China	2019		FPV-G3G	K	M	A	K	V	V	S	A	D	D	Y	Q	N	N	A
MT270585.1	China	2019		FPV-G3G	K	M	A	K	V	V	S	A	D	D	Y	Q	N	N	A
MZ442305.1	China	2018		FPV-G3G	K	M	A	K	V	V	S	A	D	D	Y	Q	N	N	A
MZ442309.1	China	2017		FPV-G3G	K	M	A	K	V	V	S	A	D	D	Y	Q	N	N	A
MZ836359.1	China	2020		FPV-G3G	K	M	A	K	V	V	S	A	D	D	Y	Q	N	N	A
MZ836360.1	China	2020		FPV-G3G	K	M	A	K	V	V	S	A	D	D	Y	Q	N	N	A
MZ836370.1	China	2020		FPV-G3G	K	M	A	K	V	V	S	A	D	D	Y	Q	N	N	A
MZ836371.1	China	2020		FPV-G3G	K	M	A	K	V	V	S	A	D	D	Y	Q	N	N	A
MZ836374.1	China	2020		FPV-G3G	K	M	A	K	V	V	S	A	D	D	Y	Q	N	N	A
MZ836375.1	China	2020		FPV-G3G	K	M	A	K	V	V	S	A	D	D	Y	Q	N	N	A
MZ913314.1	China	2021	canine	FPV-G3G	K	M	A	K	V	V	S	A	D	D	Y	Q	N	N	A
MZ913315.1	China	2019	canine	FPV-G3G	K	M	A	K	V	V	S	A	D	D	Y	Q	N	N	A
MZ913316.1	China	2019	canine	FPV-G3G	K	M	A	K	V	V	S	A	D	D	Y	Q	N	N	A
MZ913317.1	China	2019	canine	FPV-G3G	K	M	A	K	V	V	S	A	D	D	Y	Q	N	N	A
MZ913318.1	China	2019	canine	FPV-G3G	K	M	A	K	V	V	S	A	D	D	Y	Q	N	N	A
MZ913319.1	China	2019	canine	FPV-G3G	K	M	A	K	V	V	S	A	D	D	Y	Q	N	N	A
MZ836369.1	China	2020		FPV-G3	K	M	A	K	V	V	S	A	D	D	Y	Q	N	N	A
MZ442307.1	China	2018		FPV-G3	K	M	A	K	V	V	F	A	D	D	Y	Q	N	N	A
