# Supplementary material for: Prevalence and Molecular Evolution of Parvovirus in Cats in Eastern Shandong, China, between 2021 and 2022
Source: Transbound Emerg Dis. 2024 Jan 5;2024:5514806. doi: 10.1155/2024/5514806 (PMC12016963; doi:10.1155/2024/5514806)
Supplement: Supplementary 2 — Key amino-acid residues in VP2 protein of 21 parvoviruses and reference FPV/CPV-2 strains from GenBank. [file 5514806.f2.zip › Table S2b (1).pdf]

78 complete VP2 sequences of CPV-2 used in Figure 4

Genbank No./Sample	collection country	collection date	host	Genotype/Group	5	80	87	91	93	103	232	267	297	300	305	323	324	370	426	440	564	568
FJ197846.1	South Korea	2007		CPV-2	A	R	M	A	N	A	I	F	S	A	D	N	Y	Q	N	T	S	G
FJ197847.1	South Korea	2007		CPV-2	A	R	M	A	N	A	I	F	S	A	D	N	Y	Q	N	T	S	G
M38245.1	USA	1990		CPV-2	A	R	M	A	N	A	I	F	S	A	D	N	Y	Q	N	T	S	G
MH329287.1	China	2016	cat	CPV-2	A	R	M	A	N	A	I	F	A	A	D	N	Y	Q	N	T	S	G
MH329288.1	China	2016	cat	CPV-2	A	R	M	A	N	A	I	F	A	A	D	N	Y	Q	N	T	S	G
M24003.1	USA	1989		CPV-2a	A	R	L	A	N	A	I	F	S	G	Y	N	Y	Q	N	T	S	G
MN451660.1	USA	1983		CPV-2a	A	R	L	A	N	A	I	F	S	G	Y	N	Y	Q	N	T	S	G
MN451667.1	USA	2019		CPV-2a	A	R	L	A	N	A	I	F	S	G	Y	N	Y	Q	N	T	S	G
DQ340407.1	Brazil	1980		new CPV-2a	A	R	L	A	N	A	I	F	S	G	Y	N	Y	Q	N	T	S	G
FJ005252.1	Italy	2002		new CPV-2a	A	R	L	A	N	A	I	F	A	G	Y	N	Y	Q	N	T	S	G
GU362932.1	Italy	2008	cat	new CPV-2a	A	R	L	A	N	A	I	F	A	G	Y	N	Y	Q	N	T	N	A
KP090138.1	India	2014	cat	new CPV-2a	A	R	L	A	N	A	I	Y	A	G	Y	N	I	Q	N	A		
KX618915.1	Singapore	2016	cat	new CPV-2a	A	R	L	A	N	A	I	Y	A	D	Y	N	I	Q	N	A	S	G
MF001439.1	China	2016		new CPV-2a	A	R	L	A	N	A	I	Y	A	G	Y	N	I	Q	N	A	S	G
MH329283.1	China	2016	cat	new CPV-2a	A	R	L	A	N	A	I	Y	A	G	Y	N	I	Q	N	A	S	G
MH329284.1	China	2016	cat	new CPV-2a	A	R	L	A	N	A	I	Y	A	G	Y	N	I	Q	N	A	S	G
MH329285.1	China	2016	cat	new CPV-2a	A	R	L	A	N	A	I	Y	A	G	Y	N	I	Q	N	A	S	G
MK675659.1	China	2017	cat	new CPV-2a	A	R	L	A	N	A	I	Y	A	G	Y	N	I	Q	N	T	S	G
MK675660.1	China	2017	cat	new CPV-2a	A	R	L	A	N	A	I	F	A	G	Y	N	I	Q	N	T	S	G
MK675661.1	China	2017	cat	new CPV-2a	A	R	L	A	N	A	I	Y	A	G	Y	N	I	Q	N	T	S	G
MK675662.1	China	2017	cat	new CPV-2a	A	R	M	A	N	A	I	F	A	G	Y	N	I	Q	N	T	S	G
MK675663.1	China	2018	cat	new CPV-2a	A	R	L	A	N	A	I	Y	A	G	Y	N	I	Q	N	T	S	G
MK675664.1	China	2018	cat	new CPV-2a	A	R	L	A	N	A	I	F	A	G	Y	N	I	Q	N	T	S	G
MK675666.1	China	2018	cat	new CPV-2a	A	R	M	A	N	A	I	Y	A	G	Y	N	I	Q	N	T	S	G
MK675667.1	China	2018	cat	new CPV-2a	A	R	L	A	N	A	I	F	A	G	Y	N	I	Q	N	T	S	G
MN561320.1	Australia	2017		new CPV-2a	A	R	L	A	N	A	I	Y	A	G	Y	N	I	Q	N	T	N	A
MT078772.1	India	2019	cat	new CPV-2a	A	R	L	A	N	A	I	Y	A	G	Y	N	I	Q	N	T	N	A
MW017578.1	China	2017		new CPV-2a	A	R	L	A	N	A	I	Y	A	V	Y	N	I	Q	N	A	S	G
MW017604.1	China	2019		new CPV-2a	A	R	L	A	N	A	I	Y	A	G	Y	N	I	Q	N	A	S	G
MW648348.1	Brazil	2019		new CPV-2a	A	R	L	A	N	A	I	Y	A	G	Y	N	I	Q	N	T	S	G
MW648349.1	Brazil	2020		new CPV-2a	A	R	L	A	N	A	I	Y	A	G	Y	N	I	Q	N	T	S	G
MW847165.1	Italy	2016	cat	new CPV-2a	A	R	L	A	N	A	I	F	A	G	Y	N	I	Q	N	T	S	G
MW847200.1	Italy	2018	cat	new CPV-2a	A	R	L	A	N	A	I	F	A	G	Y	N	I	Q	N	T	S	G
MZ357121.1	China	2020	cat	new CPV-2a	A	R	L	A	N	A	I	Y	A	G	Y	N	I	Q	N	A	S	G
MZ442308.1	China	2018	cat	new CPV-2a	A	R	L	A	N	A	I	Y	A	G	Y	N	I	Q	N	A	S	G
MZ442310.1	China	2018	cat	new CPV-2a	A	R	L	A	N	A	I	Y	A	G	Y	N	I	Q	N	A	S	G
OK384309.1	China	2019	cat	new CPV-2a	A	R	L	G	N	A	I	F	A	S	Y	N	I	Q	N	T	S	G
OM937915.1	Egypt	2019	cat	new CPV-2a	A	R	L	A	N	A	I	Y	A	G	Y	N	I	Q	N	A	S	G
M74849.1		1995		CPV-2b	A	R	L	A	N	A	I	F	S	G	Y	N	Y	Q	D	T	S	G
U22896.1	USA	1990	cat	CPV-2b	A	R	L	A	N	A	I	F	S	G	Y	N	Y	Q	D	T	S	G
Z46651.1	Poland	1994		CPV-2b	A	R	L	A	N	A	I	F	S	G	Y	N	Y	Q	D	T	S	G
KP715703.1	Thailand	2015		new CPV-2b	A	R	L	A	N	A	I	Y	A	G	Y	N	I	Q	D	T	S	G
KR611490.1	China	2014		new CPV-2b	A	R	L	A	N	A	I	Y	A	G	Y	N	I	Q	D	A	S	G
KR611499.1	China	2014		new CPV-2b	A	R	L	A	N	A	I	Y	A	G	Y	N	I	Q	D	A	S	G
MK675657.1	China	2016	cat	new CPV-2b	A	R	L	A	N	A	I	Y	A	G	Y	N	I	Q	D	A	S	G
MK675658.1	China	2016	cat	new CPV-2b	A	R	L	A	N	A	I	Y	A	G	Y	N	I	Q	D	A	S	G
MK675665.1	China	2018	cat	new CPV-2b	A	R	M	A	N	A	I	Y	A	G	Y	N	I	Q	D	A	S	G
MT270586.1	China	2019	cat	new CPV-2b	A	R	L	A	N	A	I	Y	A	G	Y	N	I	Q	D	A	S	G
MW048563.1	China	2019		new CPV-2b	A	R	L	A	N	A	I	Y	A	G	Y	N	I	Q	D	A	S	G
MW539053.1	Turkey	2020		new CPV-2b	A	R	L	A	N	A	I	Y	A	G	Y	N	I	Q	D	A	S	G
MZ836348.1	China	2020	cat	new CPV-2b	A	R	L	A	N	A	I	Y	A	G	Y	N	I	Q	D	A	S	G
OM937914.1	Egypt	2019	cat	new CPV-2b	A	R	L	A	N	A	I	Y	A	G	Y	N	I	Q	D	A	S	G
FJ005214.1	Spain	2006		CPV-2c-1	A	R	L	A	N	A	I	F	A	G	Y	N	Y	Q	E	T	S	G
FJ005233.1	Italy	2007		CPV-2c-1	A	R	L	A	N	A	I	F	A	G	Y	N	Y	Q	E	T	S	G
FJ005245.1	Italy	2008		CPV-2c-1	A	R	L	A	N	A	I	F	A	G	Y	N	Y	Q	E	T	S	G
GU362935.1	Italy	2008	cat	CPV-2c-1	A	R	L	A	N	A	I	F	A	G	Y	N	Y	Q	E	T	S	G
HQ025913.1	Italy	2010	cat	CPV-2c-1	A	R	L	A	N	A	I	F	A	G	Y	N	Y	Q	E	T	S	G
KP682522.1	Spain	2001	cat	CPV-2c-1	A	R	L	A	N	A	I	F	A	G	Y	N	Y	Q	E	T	S	G
ON646204.1	China	2018	cat	CPV-2c-1	G	R	L	A	N	A	I	F	A	G	Y	N	Y	R	E	T	S	G
KY937651.1	China	2017		CPV-2c-2	A	R	L	A	N	A	I	Y	A	G	Y	N	I	R	E	T	S	G
KY937655.1	China	2017		CPV-2c-2	A	R	L	A	N	A	I	Y	A	G	Y	N	I	R	E	T	S	G
MH177316.1	China	2017		CPV-2c-2	G	R	L	A	N	A	I	Y	A	G	Y	N	I	Q	E	T	S	G
MH711902.1	Thailand	2016	cat	CPV-2c-2	G	R	L	A	N	A	I	Y	A	G	Y	N	I	R	E	T	S	G
MK518015.1	China	2016		CPV-2c-2	G	R	L	A	N	A	I	Y	A	G	Y	N	I	R	E	T	S	G
MT270587.1	China Beijing	2019	cat	CPV-2c-2	G	R	L	A	N	A	I	Y	A	G	Y	N	I	R	E	T	S	G
MT270588.1	China Beijing	2019	cat	CPV-2c-2	G	R	L	A	N	A	I	Y	A	G	Y	N	I	R	E	T	S	G
MT270589.1	China Beijing	2019	cat	CPV-2c-2	G	R	L	A	N	A	I	Y	A	G	Y	N	I	R	E	T	S	G
MT270590.1	China Beijing	2019	cat	CPV-2c-2	G	R	L	A	N	A	I	Y	A	G	Y	N	I	R	E	T	S	G
MT488460.1	China	2018		CPV-2c-2	A	R	L	A	N	A	I	Y	A	G	Y	N	I	R	E	T	S	G
MT488468.1	China	2017		CPV-2c-2	A	R	L	A	N	A	I	Y	A	G	Y	N	I	R	E	T	S	G
MW017601.1	China	2019		CPV-2c-2	A	R	L	A	N	A	I	Y	A	G	Y	N	I	R	E	T	S	G
MW017608.1	China	2020		CPV-2c-2	G	R	L	A	N	A	I	Y	A	G	Y	N	I	R	E	T	S	G
MZ836349.1	China Beijing	2020	cat	CPV-2c-2	G	R	L	A	N	A	I	Y	A	G	Y	N	I	R	E	T	S	G
MZ836372.1	China Beijing	2020	cat	CPV-2c-2	A	R	L	A	N	A	I	Y	A	G	Y	N	I	R	E	T	S	G
OM322821.1	China Jilin	2021	cat	CPV-2c-2	A	R	L	A	N	A	I	Y	A	G	Y	N	I	R	E	T	S	G
OM918770.1	China Jilin	2021	cat	CPV-2c-2	A	R	L	A	N	A	I	Y	A	G	Y	N	I	R	E	T	S	G
OM918781.1	China Jilin	2021	cat	CPV-2c-2	A	R	L	A	N	A	I	Y	A	G	Y	N	I	R	E	T	S	G
OQ535515/SDYT2	China	2022	cat	CPV-2c-2	A	R	L	A	N	A	I	Y	A	G	Y	N	I	R	E	T	S	G
