# Supplementary material for: Prevalence and Molecular Evolution of Parvovirus in Cats in Eastern Shandong, China, between 2021 and 2022
Source: Transbound Emerg Dis. 2024 Jan 5;2024:5514806. doi: 10.1155/2024/5514806 (PMC12016963; doi:10.1155/2024/5514806)
Supplement: Supplementary 2 — Key amino-acid residues in VP2 protein of 21 parvoviruses and reference FPV/CPV-2 strains from GenBank. [file 5514806.f2.zip › Table S2c (1).pdf]

58 partial VP2 sequences of CPV-2 (cat host)

Genbank No.	collection country	collection date	host	Genotype/Group	80	87	91	93	103	232	267	297	300	305	323	324	370	426	564	568
HM042727.1	Turkey	2009	cat	CPV-2a																
HM042728.1	Turkey	2009	cat	CPV-2a																
HM042729.1	Turkey	2009	cat	CPV-2a																
HM042730.1	Turkey	2009	cat	CPV-2a																
HM042732.1	Turkey	2009	cat	CPV-2a																
HM042733.1	Turkey	2009	cat	CPV-2a																
HM042734.1	Turkey	2010	cat	CPV-2a																
HM042741.1	Turkey	2012	cat	CPV-2a																
JX459572.1	India	2011	cat	CPV-2a							Y	A	G	Y	N	I	Q	N		
KM262072.1	Italy	2012	cat	CPV-2a							F	A	G	Y	N	Y	Q	N		
MH127882.1	China Taiwan	2015	cat	CPV-2a									G	Y	N	I	Q	N		
MH127883.1	China Taiwan	2015	cat	CPV-2a									G	Y	N	I	Q	N		
MH127884.1	China Taiwan	2016	cat	CPV-2a									G	Y	N	I	Q	N		
MH127885.1	China Taiwan	2016	cat	CPV-2a									G	Y	N	I	Q	N		
MH127886.1	China Taiwan	2017	cat	CPV-2a									G	Y	N	I	Q	N		
MH127887.1	China Taiwan	2017	cat	CPV-2a									G	Y	N	I	Q	N		
MH127888.1	China Taiwan	2017	cat	CPV-2a									G	Y	N	I	Q	N		
MH127889.1	China Taiwan	2017	cat	CPV-2a									G	Y	N	I	Q	N		
MH127890.1	China Taiwan	2017	cat	CPV-2a									G	Y	N	I	Q	N		
MH127891.1	China Taiwan	2017	cat	CPV-2a									G	Y	N	I	Q	N		
OL547659.1	China	2017-2019	cat	CPV-2a	R	M	A	N	V	V	Y	S	G	Y						
OL547672.1	China	2017-2019	cat	new CPV-2a	R	M	A	N	A	I	Y	A	G	Y						
OL547676.1	China	2017-2019	cat	CPV-2a	K	M	A	N	V	V	Y	S	G	D						
OL547679.1	China	2017-2019	cat	CPV-2a	R	L	A	N	A	I	Y	S	G	Y						
OL547699.1	China	2020	cat	CPV-2a	R	L	A	N	A	I	Y	A	G	Y						
OL547721.1	China	2020	cat	new CPV-2a																
OL989672.1	China	2021	cat	CPV-2a	R	L	A	N	A	I	Y	S	G	D	D	Y	Q	N		
OL989673.1	China	2021	cat	CPV-2a	R	L	A	N	A	I	Y	S	G	Y	N	N	Q	N		
OL989676.1	China	2021	cat	CPV-2a	R	L	A	N	A	I	Y	S	G	D	D	Y	Q	N		
OL989677.1	China	2021	cat	CPV-2a	R	L	A	N	A	I	Y	S	G	Y	N	Y	Q	N		
OL989687.1	China	2021	cat	CPV-2a	R	L	A	N	A	I	Y	A	G	Y	N	I	Q	N		
OM891547.1	India	2020	cat	CPV-2a							Y	A	G	Y	N	I	Q	N		
OQ024195.1	India	2021	cat	CPV-2a								A	G	Y	N	I	Q	H		
KP033251.1	South Africa	2014	cat	CPV-2b	R	L	A	N	A	I	F	N	G	Y	N	Y	Q	D	S	G
KM262068.1	Italy	2012	cat	CPV-2b							F	A	G	Y	N	Y	Q	D	S	G
MH127897.1	China Taiwan	2015	cat	CPV-2b									G	Y	N	Y	Q	D		
MH127898.1	China Taiwan	2015	cat	CPV-2b									G	Y	N	I	Q	D		
MH127899.1	China Taiwan	2015	cat	CPV-2b									G	Y	N	Y	Q	D		
MH127900.1	China Taiwan	2015	cat	CPV-2b									G	Y	N	Y	Q	D		
MH127901.1	China Taiwan	2015	cat	CPV-2b									G	Y	N	I	Q	D		
MH127902.1	China Taiwan	2016	cat	CPV-2b									G	Y	N	Y	Q	D		
MH127903.1	China Taiwan	2016	cat	CPV-2b									G	Y	N	Y	Q	D		
OM502014.1	Turkey	2021	cat	CPV-2b							Y	A	G	Y	N	I	Q	D		
OM805994	Turkey	2021	cat	CPV-2b								A	G	Y	N	I	Q	D		
JF280913.1	Turkey	2010	cat	CPV-2c								S	A	D	D	Y	Q	E		
KF824851.1	Portugal	2008	cat	CPV-2c														E	S	G
MG560138.1	Brazil	2016	cat	CPV-2c														E	S	G
MH127909.1	China Taiwan	2017	cat	CPV-2c									G	Y	N	I	R	E		
OL547652.1	China SiChuan	2017-2019	cat	CPV-2c	R	L	A	N	A	I	Y	A	G	D						
OL547723.1	China SiChuan	2017-2020	cat	CPV-2c	R	L	A	N	A	I	Y	A	G	Y						
OP729183.1	India	2021	cat	CPV-2c							Y	A	G	Y	N	I	Q	E		
OP729184.1	India	2021	cat	CPV-2c							Y	A	G	Y	N	I	R	E		
OP961983.1	India	2021	cat	CPV-2c							Y	A	G	Y	N	I	R	E		
OQ024194.1	India	2021	cat	CPV-2c								A	G	Y	N	I	R	Q		
OQ024196.1	India	2021	cat	CPV-2c								A	G	Y	N	I	R	E		
OQ024197.1	India	2021	cat	CPV-2c								A	G	Y	N	I	R	E		
OQ024198.1	India	2021	cat	CPV-2c								A	G	Y	N	I	R	E		
OQ024199.1	India	2021	cat	CPV-2c								A	G	Y	N	I	R	E		
