# Supplementary material for: Prevalence and Molecular Evolution of Parvovirus in Cats in Eastern Shandong, China, between 2021 and 2022
Source: Transbound Emerg Dis. 2024 Jan 5;2024:5514806. doi: 10.1155/2024/5514806 (PMC12016963; doi:10.1155/2024/5514806)
Supplement: Supplementary 3 — Detection of diarrhea-associated viruses in 51 feline fecal samples. “+” indicates positive test results. [file 5514806.f3.docx]

| **Samples** | **Virus species** | | | | | | | | | | | | |
| --- | --- | --- | --- | --- | --- | --- | --- | --- | --- | --- | --- | --- | --- |
|  | **FPV** | **FBoV** | **FBufa** | **FChPV** | **FSCV** | **FCoV** | **FCV** | **FKoV** | **FNoV** | **FAstV** | **FRV** | **CPV-2** |  |
| **SDQD5** |  |  |  | **+** |  |  |  |  |  |  |  |  |  |
| **SDQD9** | **+** | **+** |  |  |  | **+** |  |  |  |  |  |  |  |
| **SDQD12** | **+** | **+** |  |  |  | **+** |  |  |  | **+** |  |  |  |
| **SDQD14** | **+** | **+** |  |  |  | **+** |  |  |  | **+** |  |  |  |
| **SDQD23** | **+** | **+** |  |  |  | **+** |  |  |  | **+** |  |  |  |
| **SDQD24** | **+** |  |  |  |  | **+** |  |  |  |  |  |  |  |
| **SDYT27** | **+** |  |  |  |  | **+** |  |  |  |  |  |  |  |
| **SDYT28** | **+** |  |  |  |  | **+** |  |  |  | **+** |  |  |  |
| **SDYT29** | **+** |  |  |  |  |  |  | **+** |  |  |  |  |  |
| **SDYT41** | **+** |  |  |  |  |  |  |  |  | **+** |  |  |  |
| **SDYT61** |  |  |  |  |  | **+** |  |  |  |  |  |  |  |
| **SDYT1** | **+** |  |  |  |  |  |  |  |  |  |  |  |  |
| **SDYT2** |  |  |  |  |  |  |  |  |  |  |  | **+** |  |
| **SDQD6** | **+** |  |  |  |  |  |  |  |  |  |  |  |  |
| **SDQD8** | **+** |  |  |  |  |  |  |  |  |  |  |  |  |
| **SDQD13** | **+** |  |  |  |  |  |  |  |  |  |  |  |  |
| **SDQD15** | **+** |  |  |  |  |  |  |  |  |  |  |  |  |
| **SDQD19** | **+** |  |  |  |  |  |  |  |  |  |  |  |  |
| **SDQD21** | **+** |  |  |  |  |  |  |  |  |  |  |  |  |
| **SDQD22** | **+** |  |  |  |  |  |  |  |  |  |  |  |  |
| **SDYT30** | **+** |  |  |  |  |  |  |  |  |  |  |  |  |
| **SDYT39** | **+** |  |  |  |  |  |  |  |  |  |  |  |  |
| **SDYT40** | **+** |  |  |  |  |  |  |  |  |  |  |  |  |

**Table S3.** Detection of diarrhea-associated viruses in 51 feline fecal samples.
