# Supplementary figures and images for: Prevalence and Molecular Evolution of Parvovirus in Cats in Eastern Shandong, China, between 2021 and 2022
Source: Transbound Emerg Dis. 2024 Jan 5;2024:5514806. doi: 10.1155/2024/5514806 (PMC12016963; doi:10.1155/2024/5514806)

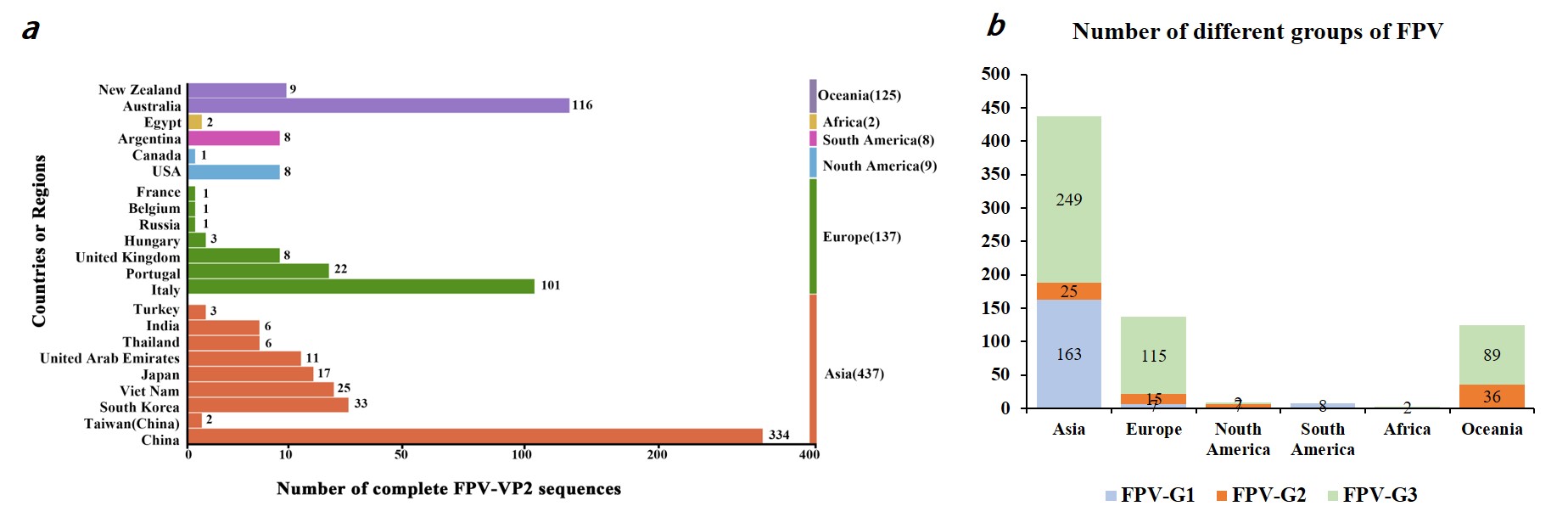

Supplement: Supplementary 4 — Distribution of complete VP2 sequences of FPV (cat/dog host). [file 5514806.f4.jpg]

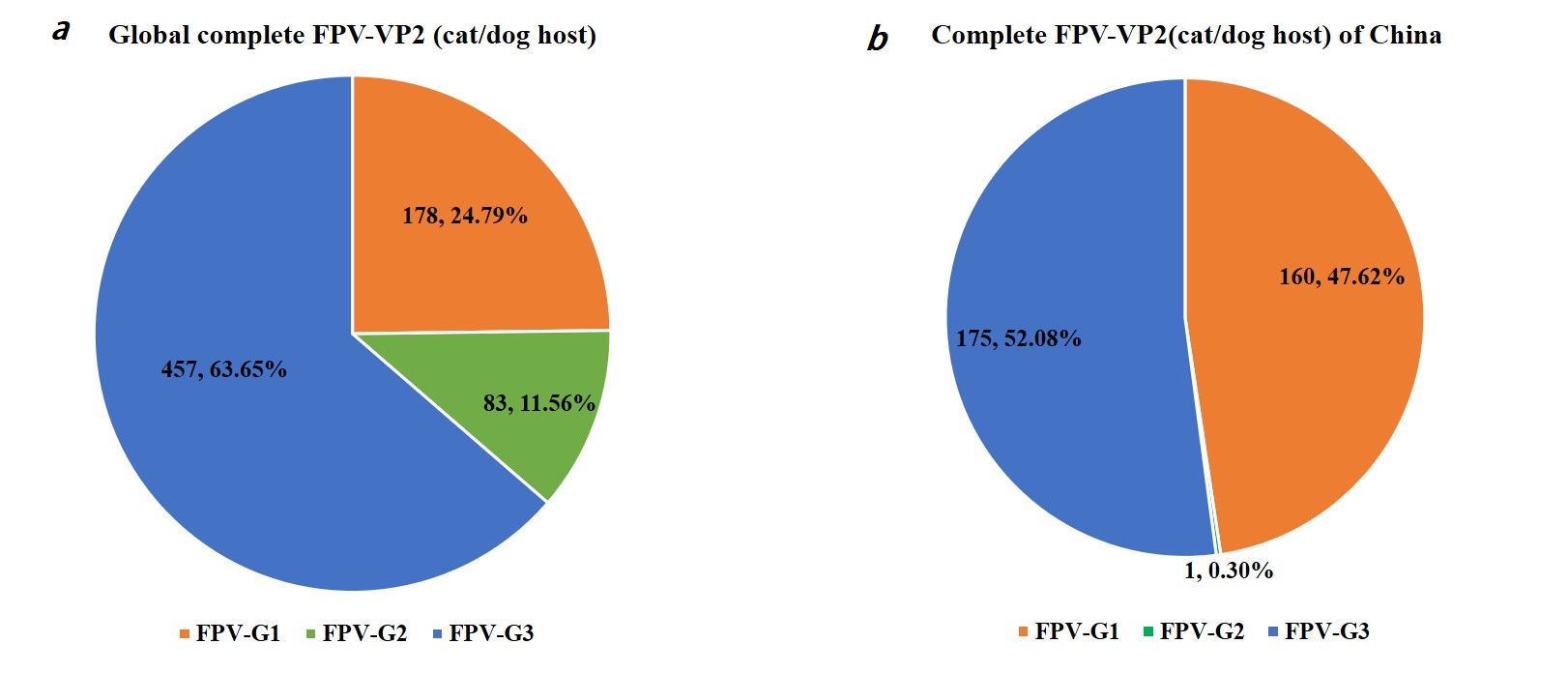

Supplement: Supplementary 5 — Quantity proportion of FPV-G1/G2/G3 groups. [file 5514806.f5.jpg]

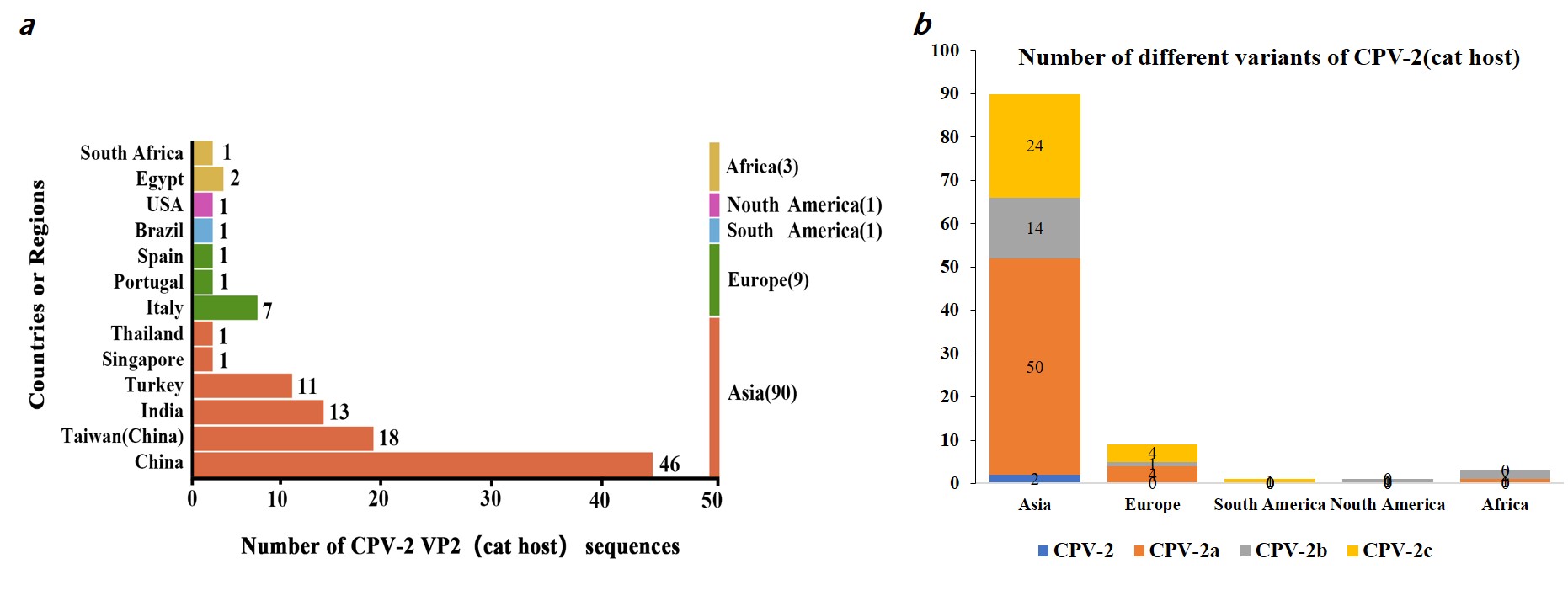

Supplement: Supplementary 6 — Distribution of CPV-2 variants (cat host). [file 5514806.f6.jpg]

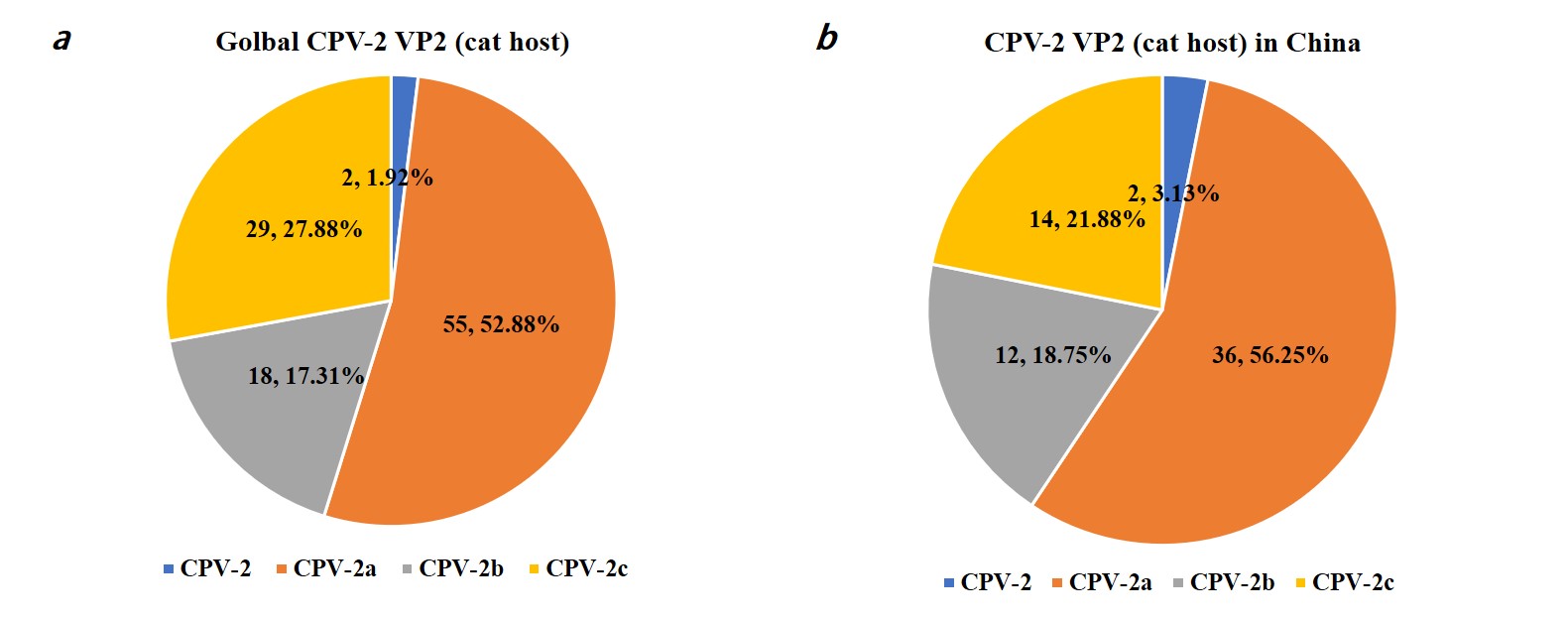

Supplement: Supplementary 7 — Quantity proportion of CPV-2/2a/2b/2c (cat host). [file 5514806.f7.jpg]
